# Chronic Caffeine Consumption, Alone or Combined with Agomelatine or Quetiapine, Reduces the Maximum EEG Peak, As Linked to Cortical Neurodegeneration, Ovarian Estrogen Receptor Alpha, and Melatonin Receptor 2

**DOI:** 10.1007/s00213-024-06619-4

**Published:** 2024-06-06

**Authors:** Sherine Abdelmissih, Sara Adel Hosny, Heba M. Elwi, Walaa Mohamed Sayed, Mohamed Ali Eshra, Olfat Gamil Shaker, Nancy F. Samir

**Affiliations:** 1 Department of Medical Pharmacology, Faculty of Medicine Kasr Al-Ainy, Cairo University, Cairo, Egypt; 2 Department of Medical Histology, Faculty of Medicine Kasr Al-Ainy, Cairo University, Cairo, Egypt; 3 Department of Medical Biochemistry and Molecular Biology, Faculty of Medicine Kasr Al-Ainy, Cairo University, Cairo, Egypt; 4 Department of Anatomy and Embryology, Faculty of Medicine Kasr Al-Ainy, Cairo University, Cairo, Egypt; 5 Department of Medical Physiology, Faculty of Medicine Kasr Al-Ainy, Cairo University, Cairo, Egypt

**Keywords:** Caffeine, Estrus cycle, Neurodegeneration, Estradiol, Antimullerian hormone, E2Rα, Agomelatine, Quetiapine, A2AR, MT2R

## Abstract

**Rationale:**

Evidence of the effects of chronic caffeine (CAFF)-containing beverages, alone or in combination with agomelatine (AGO) or quetiapine (QUET), on electroencephalography (EEG), which is relevant to cognition, epileptogenesis, and ovarian function, remains lacking. Estrogenic, adenosinergic, and melatonergic signaling is possibly linked to the dynamics of these substances.

**Objectives:**

The brain and ovarian effects of CAFF were compared with those of AGO + CAFF and QUET + CAFF. The implications of estrogenic, adenosinergic, and melatonergic signaling and the brain-ovarian crosstalk were investigated.

**Methods:**

Adult female rats were administered AGO (10 mg/kg), QUET (10 mg/kg), CAFF, AGO + CAFF, or QUET + CAFF, once daily for 8 weeks. EEG, estrous cycle progression, and microstructure of the brain and ovaries were examined. Brain and ovarian 17β-estradiol (E2), antimullerian hormone (AMH), estrogen receptor alpha (E2Rα), adenosine receptor 2A (A2AR), and melatonin receptor 2 (MT2R) were assessed.

**Results:**

CAFF, alone or combined with AGO or QUET, reduced the maximum EEG peak, which was positively linked to ovarian E2Rα, negatively correlated to cortical neurodegeneration and ovarian MT2R, and associated with cystic ovaries. A large corpus luteum emerged with AGO + CAFF and QUET + CAFF, antagonizing the CAFF-mediated increased ovarian A2AR and reduced cortical E2Rα. AGO + CAFF provoked TTP delay and increased ovarian AMH, while QUET + CAFF slowed source EEG frequency to δ range and increased brain E2.

**Conclusions:**

CAFF treatment triggered brain and ovarian derangements partially antagonized with concurrent AGO or QUET administration but with no overt affection of estrus cycle progression. Estrogenic, adenosinergic, and melatonergic signaling and brain-ovarian crosstalk may explain these effects.

**Supplementary Information:**

The online version contains supplementary material available at 10.1007/s00213-024-06619-4.

## Introduction

Caffeine (CAFF)-containing beverages, such as coffee and some carbonated beverages, are the most consumed psychostimulants. While several previous studies have focused on the cognitive-enhancing ability of CAFF (Costa et al. 2010; Ruggiero et al. 2022) by blocking adenosine receptors, some studies have reported impaired memory with CAFF consumption (Cornelis et al. 2020; Zhang et al. 2020) and limited efficacy in cognitive improvement (García et al. 2017; Zabelina & Silvia 2020). The controversial effect of CAFF on cognition extends to seizure susceptibility, with inconsistencies related to whether CAFF prevents or provokes seizures (Bauer & Sander 2019; van Koert et al. 2018).

Despite the availability of behavioral tests for cognition and seizures, electroencephalography (EEG) changes might precede overt behavioral effects, thus detecting early, asymptomatic disorders (Meghdadi et al. 2021; Snyder et al. 2011; Trambaiolli et al. 2017). Also, no single behavioral test can assess all aspects of cognition in animal models; instead, each test addresses a specific cognitive domain (Stephan et al. 2019)*.* In the recording setting used herein, scalp EEG is a noninvasive and objective tool used to detect slight cognitive effects and epileptogenesis before seizures. Indeed, EEG, a gold standard for the detection of seizure activity, can predict disorders prior to symptomatic manifestations (Benbadis et al. 2020), including epileptogenesis, a lag period from the occurrence of brain insult until the visible spontaneous seizures (Lukasiuk & Becker 2014).

In the context of cognition, adenosine, with subsequent activation of some adenosine receptor (AR) subtypes, has been shown to enhance memory and learning, support neuronal plasticity, and reduce neuronal loss. Such favorable effects are dependent on the context of the disorder (Chen 2014). Adenosine is regarded as an antiseizure molecule; however, the activation of adenosine receptor 2A (A2AR) induces seizures and is linked to memory impairment (Temido-Ferreira et al. 2020), neuroinflammation, and neuronal death (He et al. 2020). These findings necessitate further investigations regarding CAFF in terms of cognition and seizure. The notion that regular CAFF ingestion can upregulate AR in the brain (Snel & Lorist 2011) exacerbated the ambiguity of the CAFF dilemma.

Similarly, melatonin boosts cognitive performance in animal models of cognitive impairment (Yalcin et al. 2023) and in patients with Alzheimer’s disease (AD), although it impairs memory in healthy individuals (Sumsuzzman et al. 2021).

Therefore, given that CAFF reduces melatonin levels at nighttime (Shilo et al. 2002) and that melatonin receptor type 2 (MT2R) downregulation is associated with neurodegenerative changes (Savaskan et al. 2005), CAFF may mitigate cognitive performance. In addressing seizures, melatonin was deemed beneficial as an add-on therapy for epilepsy (Maghbooli et al. 2023) since low melatonin levels have been reported in patients during seizures (Jain & Besag 2013). These findings suggest that CAFF is a risk factor for seizures. The synergistic link between melatonin and adenosine in regulating sleep (Gandhi et al. 2015) attracted attention to MT2R and its link to A2AR, following the chronic consumption of insomniac CAFF drinks, in terms of EEG changes relevant to cognition and epileptogenesis.

The effects of CAFF on female fertility are enigmatic. Indeed, previous studies have reported that CAFF reduces female fertility (Hassan & Killick 2004; Wesselink et al. 2016), while in other studies, no causal relationship was found (Chavarro et al. 2009; Taylor et al. 2011). Nonetheless, CAFF consumption has been associated with a reduction of free estradiol (E2) (Choi et al. 2011; Kotsopoulos et al. 2009).

Melatonin may also be involved in female fertility since it is synthesized in situ from serotonin in the ovaries (Ezzati et al. 2021). A higher melatonin content has been reported in follicular fluid relative to blood (Itoh et al. 1997), which increases as follicles approach maturation (Reiter et al. 2014). Notably, MT2R is primarily expressed in preovulatory follicles in the granulosa and luteal cells in both humans and rats (Soares et al. 2003). In rats, melatonin disturbance affects ovarian functions (Starr 2011). Under normal E2 and antimullerian hormone (AMH) levels, estrogen receptor alpha (E2Rα) overexpression is one of the pathways triggering ovulation, aided by the recruitment of MT2R and the suppression of A2AR expression to initiate the inflammatory processes required for estrus cycle progression (Fernando & Rombauts 2014). Therefore, in this study, we assessed ovarian A2AR and MT2R.

CAFF interacts with multiple psychotropic medications, yielding pleiotropic effects. A synthetic analog of melatonin and a non-selective melatonin agonist, agomelatine (AGO), is among the currently available sleep aids with an antidepressant activity and no proven tolerance or dependence (Kasper et al. 2010). AGO might not affect serotonin receptors to the same extent as melatonin receptors, owing to its lower affinity for serotonin receptors (Descamps et al. 2009) and has been investigated as a cognitive enhancer and an antiseizure agent. Previous studies addressing the CAFF-AGO interactions remain scarce and limited to a synergistic antidepressant activity (Poleszak et al. 2016) or otherwise reduced antidepressant effect of AGO with CAFF withdrawal (Szopa et al. 2018). In terms of female reproductive functions, chronic AGO administration provokes a delayed estrus cycle progression in mice (KACAR et al. 2023); however, data on the effects of AGO on ovarian functions, in case of concurrent CAFF consumption, remain limited.

Furthermore, evidence concerning the interactions between CAFF and antipsychotics remains lacking. Existing literature has focused on the effects of CAFF on psychiatric symptoms, yielding inconclusive results (Frigerio et al. 2021; Huang & Sperlágh 2021; Peng et al. 2014; Topyurek et al. 2019). Among atypical antipsychotics, quetiapine (QUET), another sleep aid, triggers cognitive impairment in patients with dementia and bipolar disorder (Ballard et al. 2005; Harvey et al. 2007). Conversely, when compared with other antipsychotics, QUET showed some cognitive improvement in patients with schizophrenia (Biskin & Paris 2012; Soeiro‑de‑Souza et al. 2015). Considering seizure susceptibility, QUET is relatively safe compared with other antipsychotics. Previous studies have reported anti-epileptogenic activity for QUET (Gazdag et al. 2004), as well as neuroprotective and anti-aging effects owing to enhancing synthesis of the adenosine derivative, adenosine triphosphate (ATP) (M Ignacio et al. 2015). Lacking a direct affinity to melatonin receptors does not imply that such receptors are not involved in QUET-mediated actions. Indirect interactions may exist, based on the antidepressant effect of QUET in bipolar disorder, by enhancing serotonin neurotransmission (Prieto et al. 2010) and the fact that both serotonin and melatonin share the same precursor, tryptophan (Córdoba-Moreno et al. 2020).

Concerning female reproductive health, QUET is safe compared with conventional antipsychotics and risperidone (Bargiota et al. 2013). Replacing some antipsychotics with QUET has been reported to resolve menstrual irregularities (Takahashi et al. 2003). Notably, enhanced QUET antipsychotic activity is positively correlated to estrogen levels (González-Rodríguez & Seeman 2019). Nonetheless, QUET may be involved in adverse female reproductive outcomes (Bargiota et al. 2013). The presumptive link between QUET and melatonin signaling was applied using melatonin to antagonize the metabolic and endocrine adverse effects of QUET, among other atypical antipsychotics (Agahi et al. 2018; Romo‐Nava et al. 2014). Data on the effects of antipsychotics on ovarian melatonin receptor expression remain limited. Therefore, herein, we explored such interaction, providing insights into the intricate CAFF-QUET interactions.

In this study, rationalizing the effects of CAFF, AGO + CAFF, and QUET + CAFF by exploring the brain-ovarian crosstalk stems from multiple studies linking peripheral estrogenic signaling to cognition (Boyle et al. 2021; Russell et al. 2019) and seizure susceptibility (Pottoo et al. 2019; Reddy et al. 2021). Such an objective is also inspired by the established brain control over ovarian functions, such as that mediated by melatonin, which regulates reproduction in photoperiodic animals (Macchi and Bruce 2004) owing to its central gonadotropic activity (Romeu et al. 2011). Melatonin, synthesized by the pineal gland, can be taken up by the ovaries (Tamura et al. 2020). Our study explored the brain-ovarian crosstalk at multiple levels, including EEG, co-existing cortical and ovarian microstructural changes, and variations of the cortical and ovarian endocrinal milieu, elucidating possible links between them.

This study compared the effects of CAFF, AGO + CAFF, and QUET + CAFF in terms of brain EEG, relevant to cognition and epileptogenesis, as well as the brain and ovarian microstructure, and investigated brain and ovarian E2, AMH levels, and E2Rα expression and their possible involvement in such effects. The possible implication of A2AR and MT2R in such changes was considered relevant to the dynamics of administered medications and/or linked to the studied effects. Identifying potential crosstalk between the brain and ovaries will provide novel insights; hence, the pathways of CAFF interactions with psychotropic medications, namely AGO and QUET, were explored. The presumptive interactions affecting the brain and ovaries will aid healthcare professionals and policymakers in providing effective treatment as well as raise awareness among consumers regarding the consequences of combining CAFF with psychotropic medications. Moreover, the adenosinergic/melatonergic pathways in the hypothesized brain and/or ovarian effects are promising therapeutic targets. Finally, the crosstalk between the brain and the ovaries, in terms of the endocrinal milieu, will help to facilitate the applications of hormonal-based neurotherapeutics.

## Materials and methods

### Animals

Adult female *Wistar* albino rats, 7–8 months of age (200–250 g), were purchased from the Animal House of Faculty of Medicine, Kasr Al-Ainy, Cairo University and maintained (n = 8/cage) in an Acclimatization Room at the Medical Pharmacology Department, Faculty of Medicine, Kasr Al-Ainy, Cairo University, 7 days prior to experimentation, under standard conditions (24 ± 2 °C room temperature; 12-h light/dark cycle with lights on at 07:30 AM), with free access to food and water. Drug administration was initiated from 08:00 AM until approximately 12:00 PM. All experimental procedures were conducted following the European Communities Council Directive 2010/63/EU. Animal handling and experimental work were conducted according to the International Animal Ethical Guidelines of the Animal Research and Reporting of In Vivo Experiments (ARRIVE Guidelines v.2.0). All animal procedures were reviewed and approved by The Institutional Animal Care and Use Committee of Cairo University, Cairo, Egypt (CU/III/F/59/22).

### Experimental design

Adult female *Wistar* albino rats *(N* = 48*)* were equally divided into the following six groups: the control group (I), wherein rats were administered 1 mL/100 g distilled water once daily for 8 weeks; AGO (II) (Sigma Aldrich Co., St. Louis, USA), wherein rats were administered 10 mg/kg AGO once daily for 8 weeks (Lapmanee et al. 2017); QUET (III) (Sigma Aldrich Co., St. Louis, USA), wherein rats were administered 10 mg/kg QUET once daily for 8 weeks (Wang et al. 2010); CAFF group (IV), wherein rats ingested CAFF once daily for 8 weeks, as alternate-day coffee or cola, so that a rat weighing 200 g received 3.4 mL of instant coffee (Cairo, Egypt) (containing 1.32 mg CAFF), alternating daily with 6.2 mL of cola (Cairo, Egypt) (containing 0.72 mg CAFF) (Ismail et al. 2015). Both drinks were given at a standing room temperature. A 20-h abstinence from caffeine is sufficient to avoid tolerance (O’callaghan et al. 2018). AGO + CAFF (V), wherein rats were provided coffee and cola on alternate days as described above, followed by oral administration of 10 mg/kg AGO once daily for 8 weeks; QUET + CAFF (VI), wherein rats were provided coffee or cola on alternate days as described above, followed by oral administration of 10 mg/kg QUET once daily for 8 weeks. No fasting was needed before treatments.

### Electroencephalography (EEG) recordings

On the last day of the study and after drug administration, EEG recording was performed for 5 min in animals sedated via intraperitoneal (ip) administration of 350 mg/kg chloral hydrate. Rats were awake with eyes closed during the recording. Five recording subdermal pin electrodes were placed as per manufacturer’s instructions (ADInstruments, Australia). EEG was sampled at 1 kHz, with low pass filter of 100 Hz and high pass filter of 0.5 Hz. EEG recordings were performed using a BioAmplifier PowerLab data acquisition system v.8.0.8. software, and LabChart software (ADInstruments, Australia). EEG was recorded on a single channel (Channel 1: source channel) and the signals were subjected to online digital filtering to be divided into five frequency bands: gamma (γ) (30–100 Hz), beta (β) (13- < -30 Hz), alpha (α) (8–- < -12 Hz), theta (θ) (4–- < -8 Hz; sinusoidal-like waves), and delta (δ) (0.5- < -4 Hz) (Bassett et al. 2014).

EEG was subjected to off-line analysis of the maximum peak (microvolts), the average time-to-peak (TTP) (milliseconds), average cyclic amplitude (microvolts), and average cyclic frequency (Hz) of the source wave and each of the five frequency bands.

### Characterization and progression of the estrous cycle

The phases of the estrus cycle are proestrus, estrus, metestrus, and diestrus, with corresponding durations of 14 h, 24–48 h, 6–8 h, and 48–72 h, respectively (Ajayi & Akhigbe 2020). Based on a typical duration of the estrus cycle in rats of 4–5 days, prior to experimentation, rats were examined once daily for 10 days (2 consecutive estrus cycles) to ensure normal estrus cycle progression and assess the actual duration of the estrus cycle.

Only rats with normal estrus cycle progression and duration were included in the study, according to the following criteria:

- 1. Rats exhibiting proestrus and metestrus phases on the first vaginal smear, proceeding to respective estrus and diestrus phases as revealed by a second vaginal smear obtained on the following day.
- 2. Rats exhibiting estrus phase on the first vaginal smear, proceeding to metestrus phase as revealed by the second and/or third vaginal smears obtained on the next two consecutive days.
- 3. Rats exhibiting diestrus phase on the first vaginal smear, proceeding to proestrus phase as revealed by the third and/or fourth vaginal smears obtained on the third and fourth days thereafter.

Two rats with similar phases of the estrus cycle were randomly assigned to one of the six groups so that each group (n = 8) contained an equal number of rats representative of the 4 phases of the estrus cycle (each group contained an equal number of rats with similar phase and each group was a representative of the different four phases).

Following the initiation of the experiment, an assessment of estrus cycle progression was conducted on day 34 at 12:00–01:00 P.M., following treatments, once daily for 5 days. Sterile cotton-tipped swabs wetted in distilled water were quickly and gently introduced, but not too deep (5–10 mm depth) (Goldman et al. 2007) or superficial to be away from external contamination. Subsequently, the swabs were carefully rotated (one twist) against the vaginal wall. The rats were not anesthetized during smear collection. Afterward, the collected samples were placed on glass slides, dried in 70% ethanol at 37 °C, and fixed in an ethanol-ether solution (1:1) for 1 min. The unstained slides were examined under the light microscope (Marcondes et al. 2002).

Respective to the duration of different phases mentioned herein, rats fulfilling one of the following conditions were considered as having delayed estrus cycle progression:

- 1. Rats with proestrus and metestrus phases on day 34 (first vaginal smear), while the second vaginal smear revealed respective proestrus and metestrus phases on the following day (day 35).
- 2. Rats with estrus phase on day 34 (first vaginal smear), while the second and/or third vaginal smears (days 35 and/or 36) revealed estrus phase.
- 3. Rats with diestrus phase on day 34 (first vaginal smear), while the third and /or fourth vaginal smear (days 36 and/or 37) revealed diestrus phase.

Vaginal smears were withdrawn at the same time daily. The assessment was repeated three times at 5-day intervals (days 44–48 and 54–58). Vaginal smears were not withdrawn for extended durations to avoid any inflammatory or hormonal stimulation, and pseudopregnancy, owing to excessive manipulation (Goldman et al. 2007). The percentage of rats with delayed estrus cycle progression from the three repeated assessments was averaged.

### Enzyme-linked immunosorbent assay (ELISA) of free E2 and AMH levels in the brain and ovaries

Rats were euthanized by decapitation under i.v. ketamine anesthesia, following which the ovaries and brains were dissected. A 50 mg sample of each tissue per rat was homogenized in phosphate-buffered saline, centrifuged at 16,000 × *g* for 10 min at 4 °C, and the supernatant was subjected to ELISA analysis according to the manufacturer’s instructions to detect E2 (SunLong Biotech Co., Ltd.; SL0504Ra) and AMH (SunLong Biotech Co., Ltd.; SL0268Ra).

### Photomicrographs of the cerebral cortex and ovaries

Dissected brain and ovarian tissues were fixed in a 10% formalin-saline solution and processed into paraffin blocks. Next, 7-µm-thick sections were cut using a microtome and mounted on glass slides for hematoxylin and eosin (H&E) staining. Ovaries were sectioned horizontally and examined for inflammatory and cystic changes. Coronal sections of the brain were assessed for neuroinflammatory and neurodegenerative changes. As an index for neurodegeneration, the number of degenerated pyramidal cells was counted in layer 5 of the motor cortex. To do so, an Olympus light microscope (Japan) connected to a “Leica Qwin 500C” image analyzer system was used (Cambridge, UK) to examine the eight randomly selected high-power fields (× 400)/section in each group.

### Immunostaining of cortical and ovarian, E2Rα, A2AR, and MT2R

Paraffin blocks of the rat cerebral cortex and ovaries were sectioned and mounted on positively charged slides for immunostaining using rabbit monoclonal antibodies against E2R-α (ab32063, Abcam PLC, Cambridge, UK), A2AR (ab260032, Abcam), and MT2R (ab167108, Abcam). Sections were incubated overnight with two drops of the primary antibody, followed by incubation for 10 min with two drops of biotinylated goat anti-polyvalent secondary antibody, and finally with two drops of streptavidin-peroxidase for an additional 10 min. Secondary antibodies were produced by immunizing goat with rabbit IgG, yielding goat anti-rabbit IgG secondary antibodies. Diaminobenzidine (DAB) was used as a chromogen, while Meyer’s hematoxylin was used as a counterstain. The positive cortical and ovarian controls for E2Rα, A2AR, and MT2R were pituitary tissues with membranous and cytoplasmic reactions, and ovarian carcinoma sections with brownish cytoplasmic, nuclear, and membranous reactions, respectively.

## Statistical Analysis

Sample size was calculated by substitution in the following formula, to obtain (n).

$n=1+\left\{2c{\left(s\div d\right)}^{2}\right\}$ (Dell et al. 2002).

where (n) is the sample size, (c) is dependent on values chosen for significance level (α) and power (1-β), for α = 0.05 and 1-β = 0.9, c equals 10.51, (s) is the standard deviation of the variable, and (d) is the desired effect.

When applied:

$$n=1+\left\{2\times 10.5\times {\left(0.5\div 0.9\right)}^{2}\right\}= 1+6.48 =7.48 \approx 8$$

Data were coded and entered using SPSS v.28 (IBM Corp., Armonk, NY, USA) by a blinded investigator. Graphs were generated using GraphPad Prism v. 10.0.0. The pie chart and scatter plots were generated using Microsoft Excel (Microsoft Office 365). Data are presented as the mean and standard deviation for normally distributed quantitative variables, median and quartiles for non-normally distributed quantitative variables, and frequencies and relative frequencies for categorical variables. Normality was assessed using normality plots and tests (Shapiro Wilk test and Kolmogorov–Smirnov test). Pairwise comparisons were conducted using analysis of variance (ANOVA) with multiple comparisons post hoc Tuckey’s test for parametric data. The Kruskal–Wallis test with multiple comparisons post hoc Dunn’s test was conducted for non-parametric data. Fischer’s Exact test was used when the expected frequency was < 5. Relative and attributable risks were calculated using the Koopman and Newcome/Wilson methods, respectively. Correlations between quantitative variables were assessed using Spearman *rho* correlation coefficient. Differences were considered statistically significant at *p* < 0.05.

## Results

### CAFF, alone or combined with AGO or QUET, reduced the maximum EEG peak

**Fig. 1:**
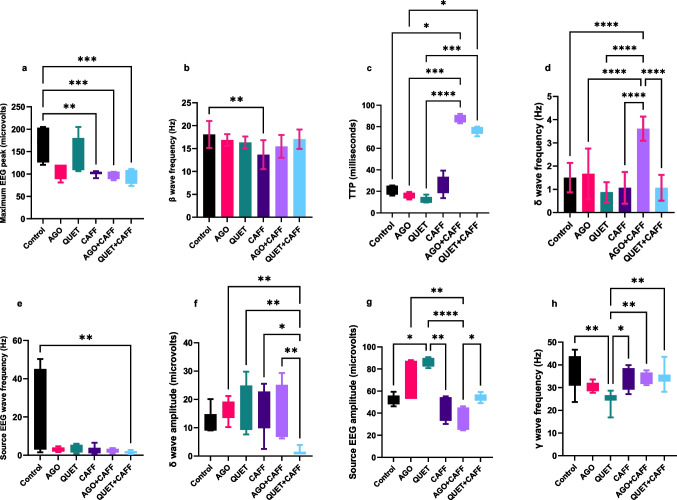
EEG analysis. **a**. Maximum EEG peak (microvolts). **b.** β wave frequency (Hz). **c.** TTP (milliseconds). **d.** δ wave frequency (Hz). **e.** Source EEG wave frequency (Hz). **f.** δ wave amplitude (microvolts). **g.** Source EEG wave amplitude (microvolts). **h.** γ wave frequency (Hz). Maximum peak, TTP, source EEG amplitude and frequency, γ wave frequency, and δ wave amplitude were analyzed by Kruskal–Wallis followed by post hoc Dunn’s test. β wave frequency and δ wave frequency were analyzed by ANOVA followed by post hoc Tukey’s. Significant when *ρ* < 0.05*; *ρ* < 0.01**; *ρ* < 0.001***; *ρ* < 0.0001****. TTP: time-to-peak. Graphs were generated using Graph Pad Prism v. 10.0.0. Adult female *Wistar* albino rats (N = 48) were equally subdivided into controls, AGO: 10 mg/kg agomelatine, oral, once daily, QUET: 10 mg/kg quetiapine, oral, once daily, CAFF: caffeine-containing beverages, as alternate-day coffee and cola, at room temperature, once daily, AGO + CAFF: caffeine-containing beverages followed by 10 mg/kg agomelatine, oral, once daily, QUET + CAFF: caffeine-containing beverages followed by 10 mg/kg quetiapine, oral, once daily. All administrations were adopted for 8 weeks

**Fig. 2:**
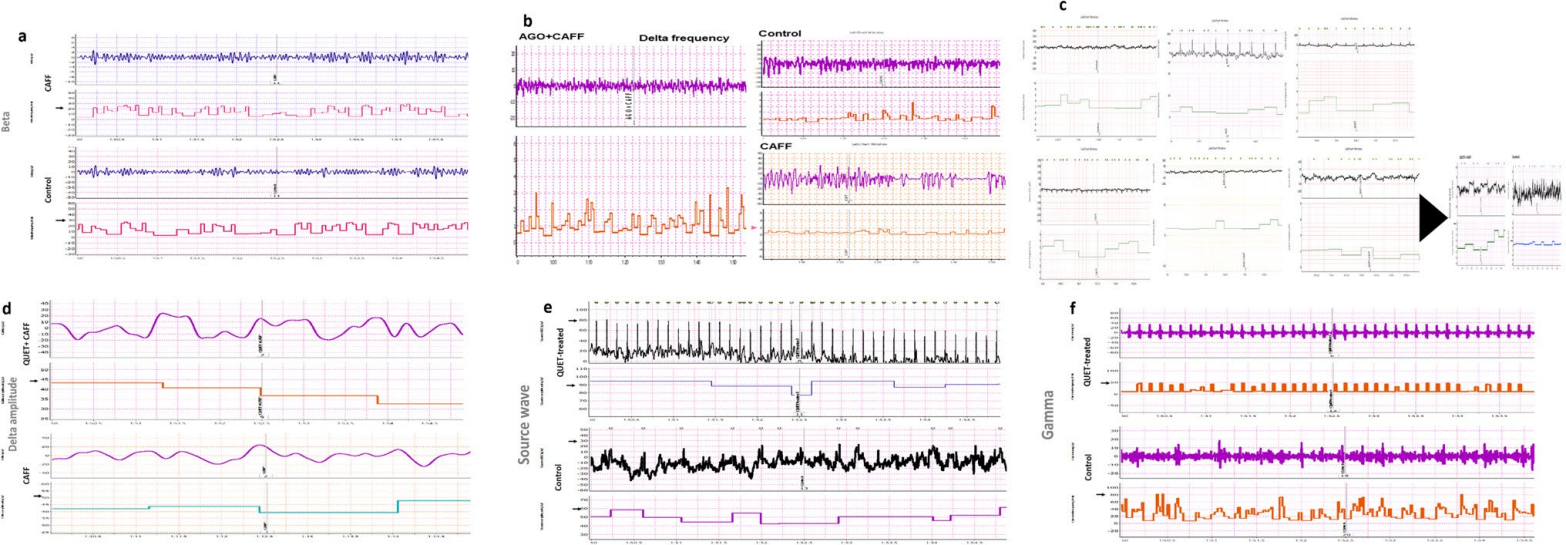
EEG tracings showing. **a**. β wave frequency of CAFF and control. **b.** δ wave frequency of AGO + CAFF, CAFF, and control. **c.** Source EEG wave frequency of studied groups, zooming in that of QUET + CAFF and control. **d.** Delta amplitude of QUET + CAFF and CAFF. **e.** Source EEG wave amplitude of QUET and control. **f.** Gamma frequency of QUET and control. Data sampling is done at 1 kHz, with low-pass filter of 100 Hz and high-pass filter of 0.5. Data acquisition is done using BioAmplifier, Power Lab system v.8.0.8., and LabChart software (*ADInstruments, Australia*). Amplitudes (microvolts) and frequency (hertz). Adult female *Wistar* albino rats (N = 48) were equally subdivided into controls, AGO: 10 mg/kg agomelatine, oral, once daily, QUET: 10 mg/kg quetiapine, oral, once daily, CAFF: caffeine-containing beverages, as alternate-day coffee and cola, at room temperature, once daily, AGO + CAFF: caffeine-containing beverages followed by 10 mg/kg agomelatine, oral, once daily, QUET + CAFF: caffeine-containing beverages followed by 10 mg/kg quetiapine, oral, once daily. All administrations were adopted for 8 weeks

Compared with the control, CAFF reduced the maximum EEG peak (microvolts) (*t*_(6)_ = 28.53*, ρ* < 0.01), which persisted with the addition of AGO or QUET (*t*_(6)_ = 28.53, *ρ* < 0.001) **(**Fig. 1a**).** There were no significant differences between CAFF and AGO + CAFF or QUET + CAFF or between AGO + CAFF and QUET + CAFF. Furthermore, CAFF significantly slowed β wave (Hz) (*F*_(5,42)_ = 3.399, *ρ* < 0.01), which was not evident with either AGO + CAFF or QUET + CAFF **(**Figs. 1b and 2a**)**. AGO + CAFF provoked EEG changes, which were not evident with CAFF, including a significantly delayed TTP (milliseconds) (*t*_(6)_ = 41.18, *ρ* < 0.05) **(**Fig. 1c**)** and a prominent increase in δ wave frequency (Hz) (*F*_(5,42)_ = 17.52, *ρ* < 0.0001), the latter exceeding that of CAFF (*F*_(5,42)_ = 17.52, *ρ* < 0.0001) **(**Figs. 1d and 2b**)**. Compared with the control, QUET + CAFF significantly slowed source EEG frequency (Hz), attaining a δ frequency range (*t*_(6)_ = 13.27, *ρ* < 0.01) **(**Figs. 1e and 2c**)**, which was not observed with CAFF. QUET + CAFF reduced the δ wave amplitude (microvolts) compared with CAFF (*t*_(6)_ = 21.13, *ρ* < 0.05) **(**Figs. 1f and 2d**)**; however, it did not significantly differ from the control.

Unlike AGO monotherapy, which did not provoke significant EEG changes compared with the control, QUET significantly increased the source EEG amplitude (microvolts) (*t*_(6)_ = 33.28, *ρ* < 0.05) **(**Figs. 1g and 2e**)** and slowed γ frequency down to the β frequency range (Hz) (*t*_(6)_ = 22.00, *ρ* < 0.01) **(**Figs. 1h and 2f**)**. Compared with the control, CAFF, whether alone or combined with AGO or QUET, did not significantly affect the amplitudes of γ, β, α, or θ EEG waves (microvolts) (Suppl. Figure 1). Neither α nor θ wave frequency (Hz) was affected in all groups.

### CAFF, alone or combined with AGO or QUET, exhibited cortical neurodegenerative changes

**Fig. 3:**
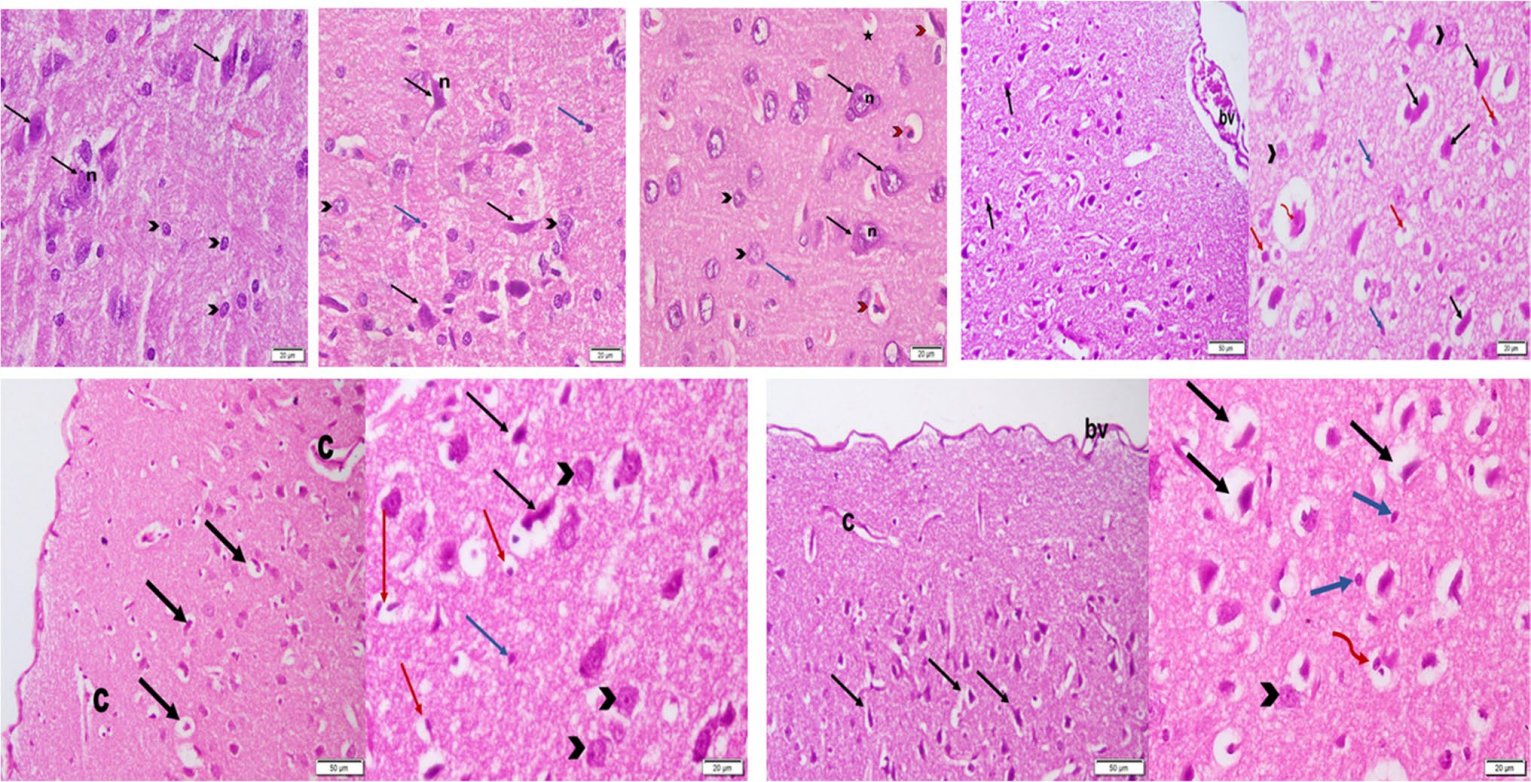
Photomicrographs of H&E-stained sections of cerebral cortex of adult female *Wistar* albino rats. As ordered from left to right over two rows, **a.** Control, showing the basophilic cytoplasm and pale vesicular nuclei (n) of pyramidal cells (black arrows). Granular cells are also present (black arrow heads) (× 400); **b.** AGO, showing, in addition to the pyramidal cells with basophilic cytoplasm (black arrows) and pale vesicular nuclei (n), and the granular cells (black arrow heads), glial cells with small dense nuclei (blue arrows) (× 400); **c.** QUET, displaying pyramidal cells (black arrows) with pale vesicular nuclei (n), granular cells (black arrow heads), glial cells (blue arrow), in addition to degenerated shrunken cells surrounded by a pericellular halo (red arrow heads) (× 400); **d.** CAFF, displaying degenerated pyramidal cells with pericellular halo and dark nuclei (black arrows), together with dilated congested blood vessels (bv) in the intermediate lamella of the pia matter (× 200); **e.** CAFF, showing multinucleated cells (red wavy arrow), granular cells (black arrowheads), glial cells with small dense nuclei (blue arrows), as well as pyknotic cells surrounded by pericellular halo (black arrows) (× 400); **f.** AGO + CAFF, showing degenerated pyramidal cells with pericellular halo and dark nuclei (black arrows), and dilated capillaries (c) in the intermediate lamella of the pia matter (× 200). **g.** AGO + CAFF, showing pyknotic cells with pericellular halo (red arrows), granular cells (black arrowheads) and glial cells with small dense nuclei (blue arrow) (× 400); **h.** QUET + CAFF, displaying degenerated pyramidal cells with pericellular halo and dark nuclei (black arrows), dilated blood vessels (bv) and capillaries (c) in intermediate lamella of the pia matter (× 200) and **i.** QUET + CAFF, showing degenerated pyramidal cells with pericellular halo and dark nuclei (black arrows), granular cells (black arrowhead) and glial cells with small dense nuclei (blue arrows), with the multinucleated cells (red wavy arrow) (× 400). Adult female *Wistar* albino rats (N = 48) were equally subdivided into controls, AGO: 10 mg/kg agomelatine, oral, once daily, QUET: 10 mg/kg quetiapine, oral, once daily, CAFF: caffeine-containing beverages, as alternate-day coffee and cola, at room temperature, once daily, AGO + CAFF: caffeine-containing beverages followed by 10 mg/kg agomelatine, oral, once daily, QUET + CAFF: caffeine-containing beverages followed by 10 mg/kg quetiapine, oral, once daily. All administrations were adopted for 8 weeks

In the AGO group, apart from the pyramidal and granular cells observed in control rats **(**Fig. 3a**)**, glial cells were observed **(**Fig. 3b**)**. Similarly, with QUET monotherapy, the cerebral cortex of rats showed glial cells as with AGO monotherapy. Scarce degenerated and shrunken pyramidal cells surrounded by pericellular halos were evident **(**Fig. 3c**)**.

The cerebral cortex of rats administered CAFF exhibited degenerated pyramidal cells, pyknotic cells, multinucleated cells, and dilated congested blood vessels **(**Figs. 3d and 3e**)**. The AGO + CAFF group exhibited cortical changes as was with CAFF, but with no obvious glial or multinucleated cells **(**Figs. 3f and 3g**)**. QUET + CAFF triggered similar cortical changes to those with CAFF **(**Figs. 3h and 3i**)**.

### CAFF, alone or combined with AGO or QUET, increased the number of degenerated pyramidal cells

**Fig. 4:**
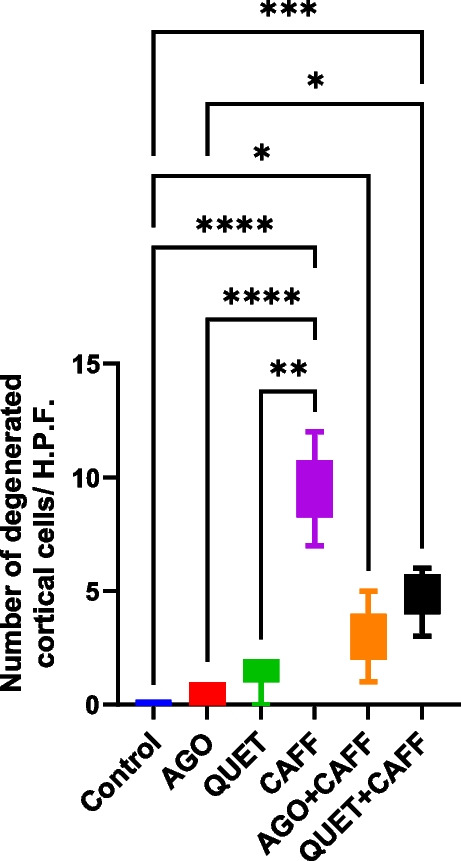
Number of degenerated pyramidal cells /HPF (× 400). Data are analyzed using Kruskal–Wallis and post hoc Dunn’s test and are represented as median and interquartile range. Significant when *ρ* < 0.05*; *ρ* < 0.01**; *ρ* < 0.001***; *ρ* < 0.0001****. The graph is generated using Graph Pad Prism v. 10.0.0. H.P.F.: high-power field. Adult female *Wistar* albino rats (N = 48) were equally subdivided into controls, AGO: 10 mg/kg agomelatine, oral, once daily, QUET: 10 mg/kg quetiapine, oral, once daily, CAFF: caffeine-containing beverages, as alternate-day coffee and cola, at room temperature, once daily, AGO + CAFF: caffeine-containing beverages followed by 10 mg/kg agomelatine, oral, once daily, QUET + CAFF: caffeine-containing beverages followed by 10 mg/kg quetiapine, oral, once daily. All administrations were adopted for 8 weeks

To illustrate the extent of neurodegenerative changes, despite the degenerated pyramidal cells observed with QUET monotherapy, the number was not significantly different from that of the control. CAFF significantly increased the number of degenerated pyramidal cells (*t*_(6)_ = 42.32, *ρ* < 0.0001) **(**Fig. 4**)**. When CAFF was administered with AGO or QUET, the number of degenerated pyramidal cells remained significantly higher than the control (*t*_(6)_ = 42.32, *ρ* < 0.05*; t*_(6)_ = 42.32, *ρ* < 0.001, respectively)** (**Fig. 4**).**

### The number of degenerated cortical cells was negatively correlated to the maximum EEG peak

The number of degenerated cortical cells was negatively correlated to the maximum EEG peak (*r* = **-0.599**, *ρ* < 0.0001) and positively to TTP (*r* = 0.450, *ρ* = 0.0013). Moreover, the number of degenerated cortical cells was negatively correlated to source EEG frequency (*r* = -0.494, *ρ* = 0.0004).

### QUET, more than AGO, antagonized the CAFF-reduced cortical E2Rα

**Fig. 5:**
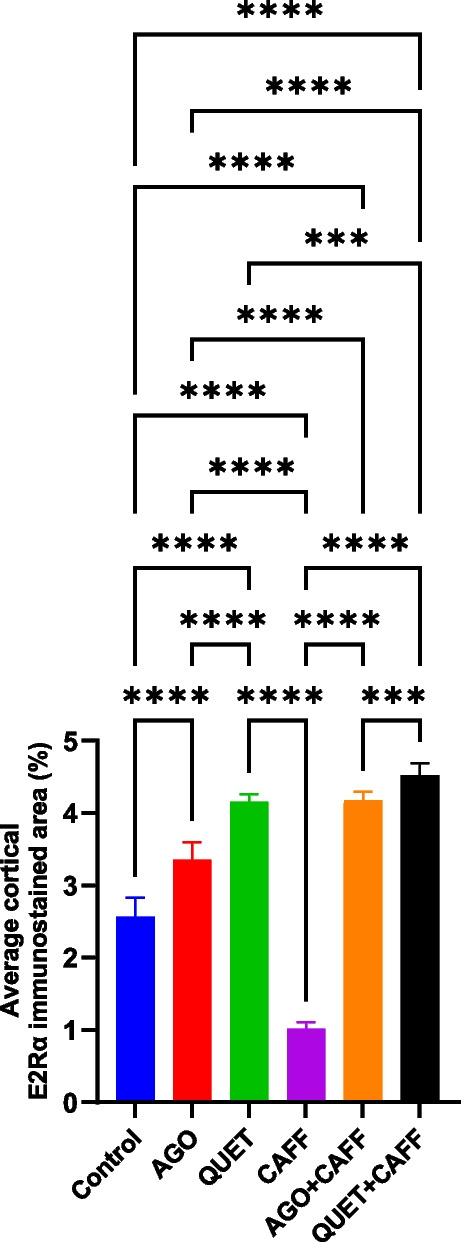
E2Rα immunoreactive area in rat cerebral cortex (percent). Data are analyzed using ANOVA, followed by post hoc Tukey’s test, and are represented as mean ± standard deviation. Graphs are generated using Graph Pad Prism v.10.0.0. *ρ* < 0.05*; *ρ* < 0.01**; *ρ* < 0.001***; *ρ* < 0.0001****. E2Rα: estrogen receptor alpha. Adult female *Wistar* albino rats (N = 48) were equally subdivided into controls, AGO: 10 mg/kg agomelatine, oral, once daily, QUET: 10 mg/kg quetiapine, oral, once daily, CAFF: caffeine-containing beverages, as alternate-day coffee and cola, at room temperature, once daily, AGO + CAFF: caffeine-containing beverages followed by 10 mg/kg agomelatine, oral, once daily, QUET + CAFF: caffeine-containing beverages followed by 10 mg/kg quetiapine, oral, once daily. All administrations were adopted for 8 weeks

Compared with the control, CAFF reduced the cortical E2Rα- immunoreactive area (*F*_(5,53)_ = 555.1, *ρ* < 0.0001) (Fig. 5 and Suppl. Figure 2); however, neither E2 nor AMH level was significantly changed. AGO antagonized the CAFF effect on cortical E2Rα-immunoreactive area, which significantly increased relative to CAFF, even exceeding that of the control (*F*_(5,53)_ = 555.1, *ρ* < 0.0001) (Fig. 5 and Suppl. Figure 2). Similarly, QUET antagonized the CAFF effect on cortical E2Rα- immunoreactive area, yielding a significantly higher value relative to CAFF, even exceeding the control (*F*_(5,53)_ = 555.1, *ρ* < 0.0001) (Fig. 5 and Suppl. Figure 2).

**Fig. 6:**
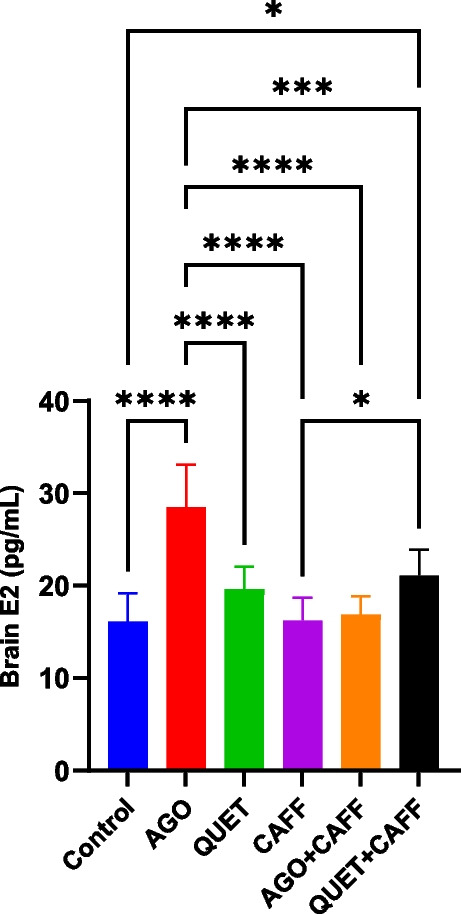
Brain E2 (pg/mL). Data are analyzed using ANOVA, followed by post hoc Tukey’s test, and are represented as mean ± standard deviation (SD). *ρ* < 0.05*; *ρ* < 0.001***. The graph is generated using Graph Pad Prism v.10.0.0. E2: estradiol. Adult female *Wistar* albino rats (N = 48) were equally subdivided into controls, AGO: 10 mg/kg agomelatine, oral, once daily, QUET: 10 mg/kg quetiapine, oral, once daily, CAFF: caffeine-containing beverages, as alternate-day coffee and cola, at room temperature, once daily, AGO + CAFF: caffeine-containing beverages followed by 10 mg/kg agomelatine, oral, once daily, QUET + CAFF: caffeine-containing beverages followed by 10 mg/kg quetiapine, oral, once daily. All administrations were adopted for 8 weeks

In this context, QUET + CAFF significantly exceeded the effect of AGO + CAFF (*F*_(5,53)_ = 555.1, *ρ* < 0.001) (Fig. 5 and Suppl. Figure 2). QUET + CAFF significantly increased brain E2 compared with the control (*F*_(5,42)_ = 19.91, *ρ* < 0.05) **(**Fig. 6**)**. The latter effect was not observed following administration of CAFF, AGO + CAFF, or QUET monotherapy. Neither AGO + CAFF nor QUET + CAFF significantly changed brain AMH levels (Suppl. Figure 3).

In contrast to CAFF, AGO and QUET monotherapies significantly increased the cortical E2Rα- immunoreactive areas relative to the control (*F*_(5,53)_ = 555.1, *ρ* < 0.0001), with QUET having a more significant effect than AGO (*F*_(5,53)_ = 555.1, *ρ* < 0.0001) (Fig. 5 and Suppl. Figure 2). The increasing effects of AGO and QUET monotherapies over cortical E2Rα-immunoreactive areas were significantly lower than those exerted when each monotherapy was combined with CAFF (*F*_(5,53)_ = 555.1, *ρ* < 0.0001, *ρ* < 0.001, respectively**)**. Brain E2 was significantly increased with AGO, but not QUET, monotherapy (*F*_(5,42)_ = 19.91, *ρ* < 0.0001) **(**Fig. 6**)**, which was abolished when AGO was co-administered with CAFF. The increase in brain E2 observed with AGO monotherapy exceeded that of QUET + CAFF (*F*_(5,53)_ = 555.1, *ρ* < 0.001). Correlations between brain E2, AMH, and cortical E2Rα are illustrated in Suppl. Figure 4.

### Some of the EEG aspects were correlated to brain E2 and cortical E2Rα

**Fig. 7:**
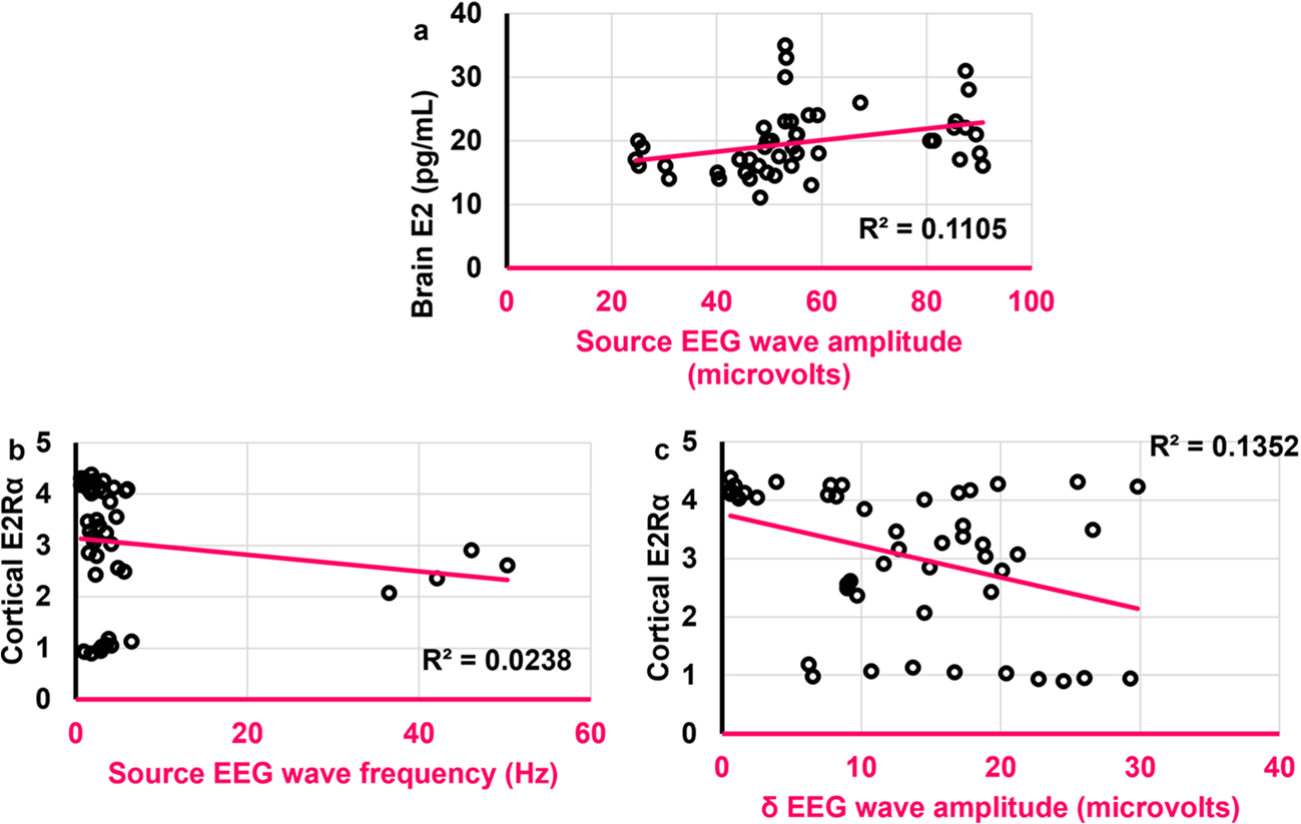
Scatter plots illustrating correlations between **a.** Source EEG amplitude (microvolts) and brain E2 (pg/mL), **b.** Source EEG frequency (Hz) and cortical E2Rα, and **c.** δ wave amplitude (microvolts) and cortical E2Rα. Scatter plots are generated using Microsoft Excel (Microsoft Office 365). Spearman *rho* correlation. Significant when *ρ* < 0.05. E2: estradiol; E2Rα: estrogen receptor alpha. Adult female *Wistar* albino rats (N = 48) were equally subdivided into controls, AGO: 10 mg/kg agomelatine, oral, once daily, QUET: 10 mg/kg quetiapine, oral, once daily, CAFF: caffeine-containing beverages, as alternate-day coffee and cola, at room temperature, once daily, AGO + CAFF: caffeine-containing beverages followed by 10 mg/kg agomelatine, oral, once daily, QUET + CAFF: caffeine-containing beverages followed by 10 mg/kg quetiapine, oral, once daily. All administrations were adopted for 8 weeks

While the amplitude of source EEG wave was positively correlated to brain E2 (*r* = 0.482,* ρ* = 0.001), the frequency of source EEG wave was negatively correlated to cortical E2Rα (*r* = -0.317,* ρ* = 0.028) **(**Fig. 7**)**. Moreover, δ wave amplitude was negatively correlated to cortical E2Rα (*r* = -0.315,* ρ* = 0.029) **(**Fig. 7**)**. The number of degenerated cortical cells was not correlated to either brain E2, AMH, or cortical E2Rα.

### Both AGO and QUET antagonized CAFF-increased cortical A2AR

**Fig. 8:**
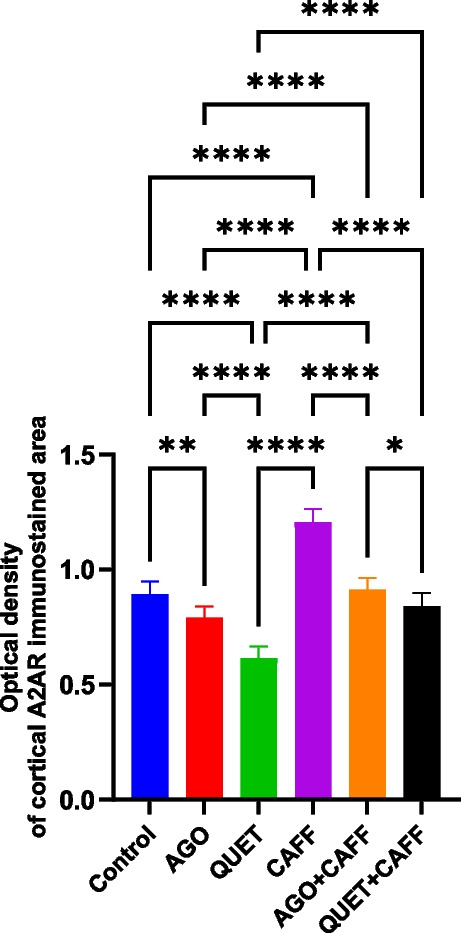
Optical density of A2AR immunoreactivity in rat cerebral cortex. Data are analyzed using ANOVA, followed by post hoc Tukey’s test, and are represented as mean ± standard deviation. Graphs are generated using Graph Pad Prism v.10.0.0. *ρ* < 0.05*; *ρ* < 0.01**; *ρ* < 0.001***; *ρ* < 0.0001****. A2AR: adenosine receptor 2A. Adult female *Wistar* albino rats (N = 48) were equally subdivided into controls, AGO: 10 mg/kg agomelatine, oral, once daily, QUET: 10 mg/kg quetiapine, oral, once daily, CAFF: caffeine-containing beverages, as alternate-day coffee and cola, at room temperature, once daily, AGO + CAFF: caffeine-containing beverages followed by 10 mg/kg agomelatine, oral, once daily, QUET + CAFF: caffeine-containing beverages followed by 10 mg/kg quetiapine, oral, once daily. All administrations were adopted for 8 weeks

CAFF significantly increased the optical density of cortical A2AR immunoreactivity compared with the control (*F*_(5,54)_ = 129.0, *ρ* < 0.0001) (Fig. 8 and Suppl. Figure 5). Both AGO and QUET antagonized the CAFF effect over the optical density of cortical A2AR immunoreactivity, as evidenced by the significant reductions relative to CAFF (*F*_(5,54)_ = 129.0, *ρ* < 0.0001) (Fig. 8 and Suppl. Figure 5), restoring them to control levels, with AGO + CAFF having a more significant effect than QUET + CAFF (*F*_(5,54)_ = 129.0, *ρ* < 0.05).

AGO and QUET monotherapies significantly reduced the optical density of cortical A2AR immunoreactivity relative to the control (*F*_(5,54)_ = 129.0, *ρ* < 0.01 and* ρ* < 0.0001, respectively), with QUET having a more significant effect than AGO (*F*_(5,54)_ = 129.0, *ρ* < 0.0001) (Fig. 8 and Suppl. Figure 5).

### Only when combined with AGO or QUET, did CAFF increase cortical MT2R

**Fig. 9:**
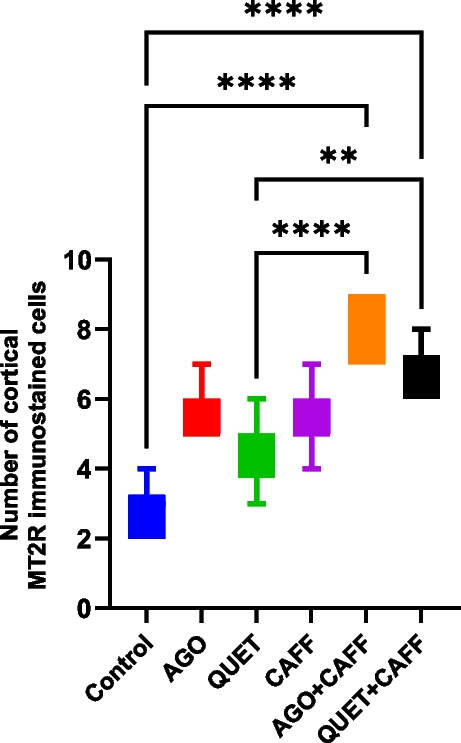
Number of MT2R immunoreactive cells in rat cerebral cortex. Data are analyzed using Kruska-Wallis, followed by post hoc Dunn’s test, and are represented as median and interquartile range. Graphs are generated using Graph Pad Prism v.10.0.0. *ρ* < 0.05*; *ρ* < 0.01**; *ρ* < 0.001***; *ρ* < 0.0001****. MT2R: melatonin receptor 2. Adult female *Wistar* albino rats (N = 48) were equally subdivided into controls, AGO: 10 mg/kg agomelatine, oral, once daily, QUET: 10 mg/kg quetiapine, oral, once daily, CAFF: caffeine-containing beverages, as alternate-day coffee and cola, at room temperature, once daily, AGO + CAFF: caffeine-containing beverages followed by 10 mg/kg agomelatine, oral, once daily, QUET + CAFF: caffeine-containing beverages followed by 10 mg/kg quetiapine, oral, once daily. All administrations were adopted for 8 weeks

CAFF did not significantly affect the number of MT2R-immunoreactive cortical cells. Unlike CAFF, AGO + CAFF and QUET + CAFF significantly increased the number of MT2R immunoreactive cortical cells relative to the control (*t*_(6)_ = 48.60, *ρ* < 0.0001) (Fig. 9 and Suppl. Figure 6); however, they showed no significant difference between them.

Like CAFF, neither AGO nor QUET affected the number of MT2R-immunoreactive cortical cells compared with the control. No significant correlation was found between cortical A2AR and cortical MT2R.

### TTP was positively correlated to cortical A2AR

**Fig. 10:**
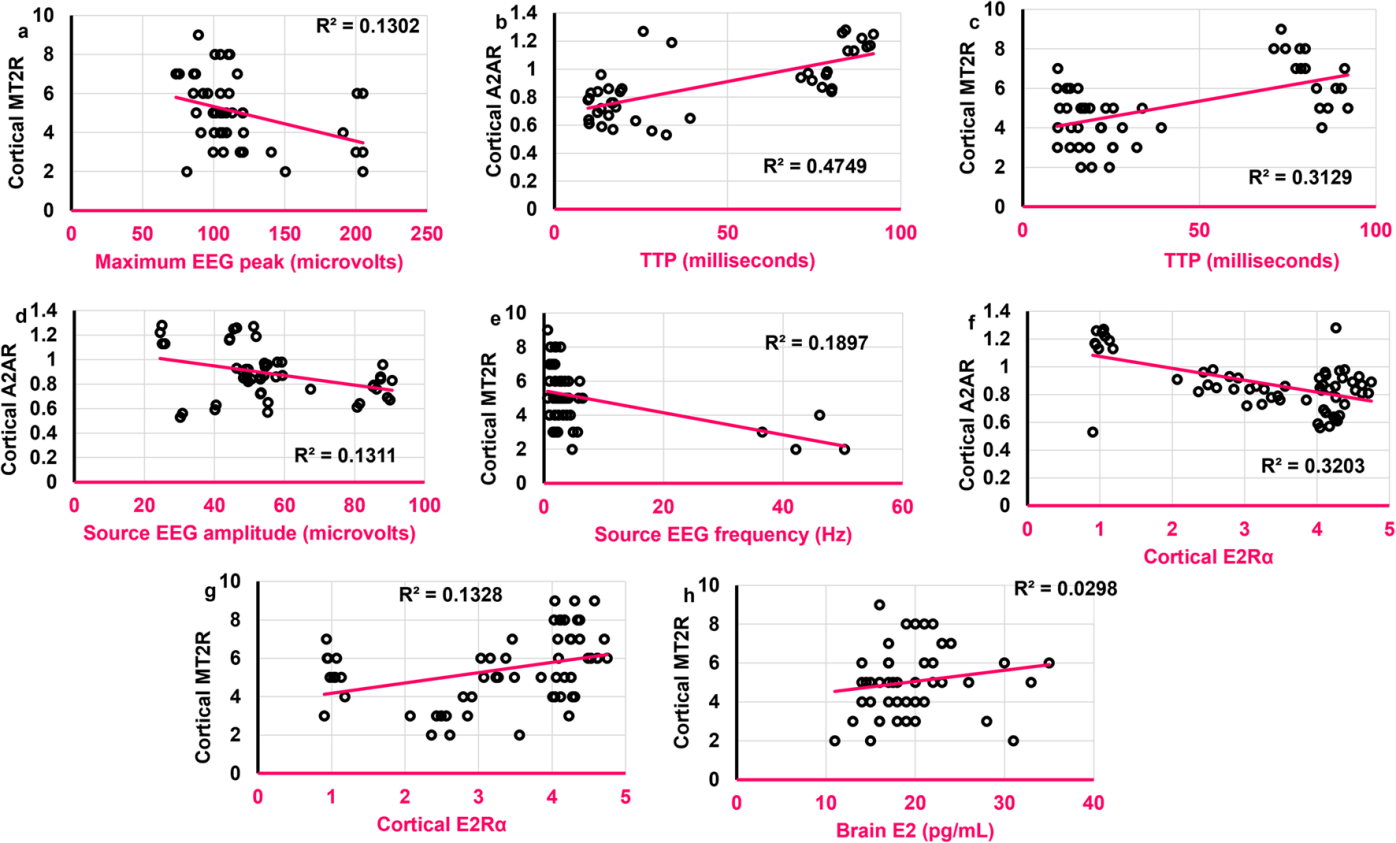
Scatter plots illustrating the correlations between **a**. Maximum EEG peak (microvolts) and cortical MT2R. **b.** TTP (milliseconds) and cortical A2AR. **c.** TTP (milliseconds) and cortical MT2R. **d.** Source EEG amplitude (microvolts) and cortical A2AR. **e.** Source EEG frequency (Hz) and cortical MT2R. **f.** Cortical E2Rα and cortical A2AR. **g.** Cortical E2Rα and cortical MT2R. **h.** Brain E2 (pg/mL) and cortical MT2R. Scatter plots are generated using Microsoft Excel (Microsoft Office 365). Spearman *rho* correlation. Significant when *ρ* < 0.05. A2AR: Adenosine receptor 2A; MT2R: Melatonin receptor 2; E2: estrogen; E2Rα: estrogen receptors alpha. Adult female *Wistar* albino rats (N = 48) were equally subdivided into controls, AGO-treated: 10 mg/kg agomelatine, oral, once daily, QUET-treated: 10 mg/kg quetiapine, oral, once daily, CAFF: caffeine-containing beverages, as alternate-day coffee and cola, at room temperature, once daily, AGO + CAFF: caffeine-containing beverages followed by 10 mg/kg agomelatine, oral, once daily, QUET + CAFF: caffeine-containing beverages followed by 10 mg/kg quetiapine, oral, once daily. All administrations were given for 8 weeks

The maximum EEG peak was negatively correlated to cortical MT2R (*r* = -0.384,* ρ* = 0.007) **(**Fig. 10a**)**, while TTP was positively correlated to both cortical A2AR (*r* = **0.652**,* ρ* < 0.0001) and cortical MT2R (*r* = 0.336,* ρ* < 0.05) (Figs. 10 b and c). The amplitude of source EEG wave was negatively correlated to cortical A2AR (*r* = -0.327,* ρ* = 0.023) **(**Fig. 10d**)**. The frequency of source EEG wave was negatively correlated to cortical MT2R (*r* = -0.381,* ρ* = 0.008) **(**Fig. 10e**)**. Concerning endocrinal correlations, cortical A2AR was negatively correlated to cortical E2Rα (*r* = -0.336,* ρ* = 0.009) **(**Fig. 10f**)**. In contrast, cortical MT2R was positively correlated to both cortical E2Rα (*r* = 0.446,* ρ* < 0.001) and brain E2 (*r* = 0.286,* ρ* = 0.049) **(**Figs. 10g and h**)**. Interestingly, the number of degenerated cortical cells was positively correlated to cortical MT2R (*r* = 0.399,* ρ* = 0.005) but was not significantly correlated to cortical A2AR.

### CAFF, alone or combined with either AGO or QUET, did not affect estrus cycle progression

While 38% of rats administered CAFF exhibited delayed estrus cycle progression, predominantly in the proestrus and estrus phases of the cycle, this was not significant compared with the control, nor was there a causal relationship (relative risk (RR) = 0.00). The risk of affection due to CAFF exposure, identified as attributable risk (AR), was 0.38. A total of 25% of rats administered AGO + CAFF or QUET + CAFF exhibited delayed estrus cycle progression, predominantly in metestrus and diestrus phases; however, this was not significantly different compared with either CAFF or control. The RR and AR were 1.5 and 0.13, respectively.

**Fig. 11:**
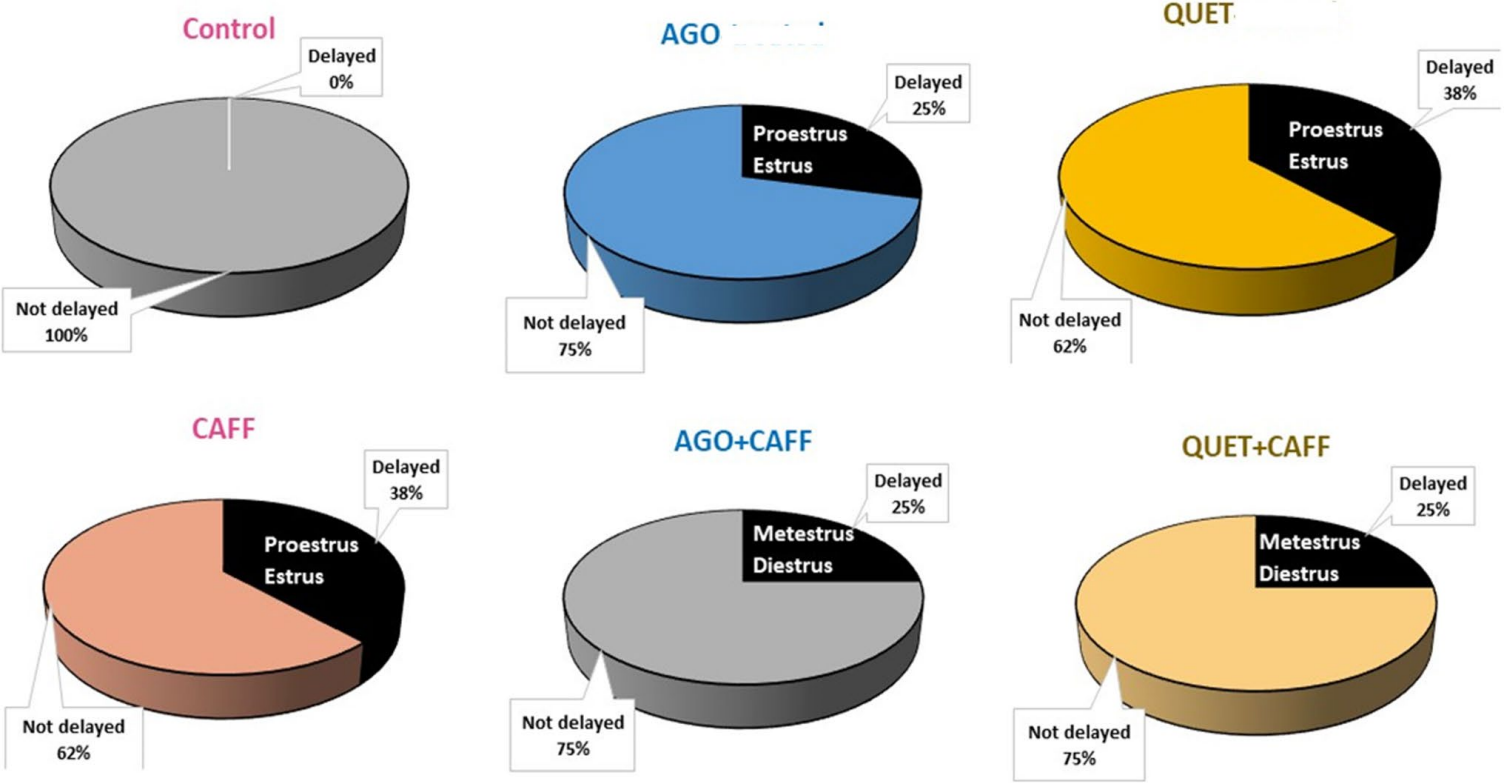
Estrus cycle progression in control, AGO (daily oral 10 mg/kg, 8 weeks), QUET (daily oral 10 mg/kg, 8 weeks), CAFF (alternate day coffee and cola at room temperature, 8 weeks), AGO + CAFF (CAFF followed by 10 mg/kg oral AGO), and QUET + CAFF (CAFF followed by 10 mg/kg oral QUET). Data are presented as the average percent of delayed estrus cycle progression over three assessments. (n = 8 per group). Data analysis was done using Fischer’s exact test. Significant when *ρ* < 0.05. The pie chart was generated using Microsoft Excel (Microsoft Office 365). Adult female *Wistar* albino rats (N = 48) were equally subdivided into controls, AGO: 10 mg/kg agomelatine, oral, once daily, QUET: 10 mg/kg quetiapine, oral, once daily, CAFF: caffeine-containing beverages, as alternate-day coffee and cola, at room temperature, once daily, AGO + CAFF: caffeine-containing beverages followed by 10 mg/kg agomelatine, oral, once daily, QUET + CAFF: caffeine-containing beverages followed by 10 mg/kg quetiapine, oral, once daily. All administrations were adopted for 8 weeks

A total of 25% and 38% of rats receiving respective AGO and QUET monotherapy showed delayed estrus cycle progression, involving, chiefly, proestrus, and estrus phases. Nevertheless, these changes were not significant compared with the control. No evident causal relationship was detected (RR = 0.00). AR for AGO and QUET monotherapies were 0.25 and 0.38, respectively. Figure 11 illustrates the average percent of delayed estrus cycle progression and the prolonged phases in the studied groups. The four phases of the estrus cycle in rats are illustrated in Suppl. Figure 7.

### CAFF combined with AGO or QUET was associated with cystic ovaries and a large *corpus* luteum

**Fig. 12:**
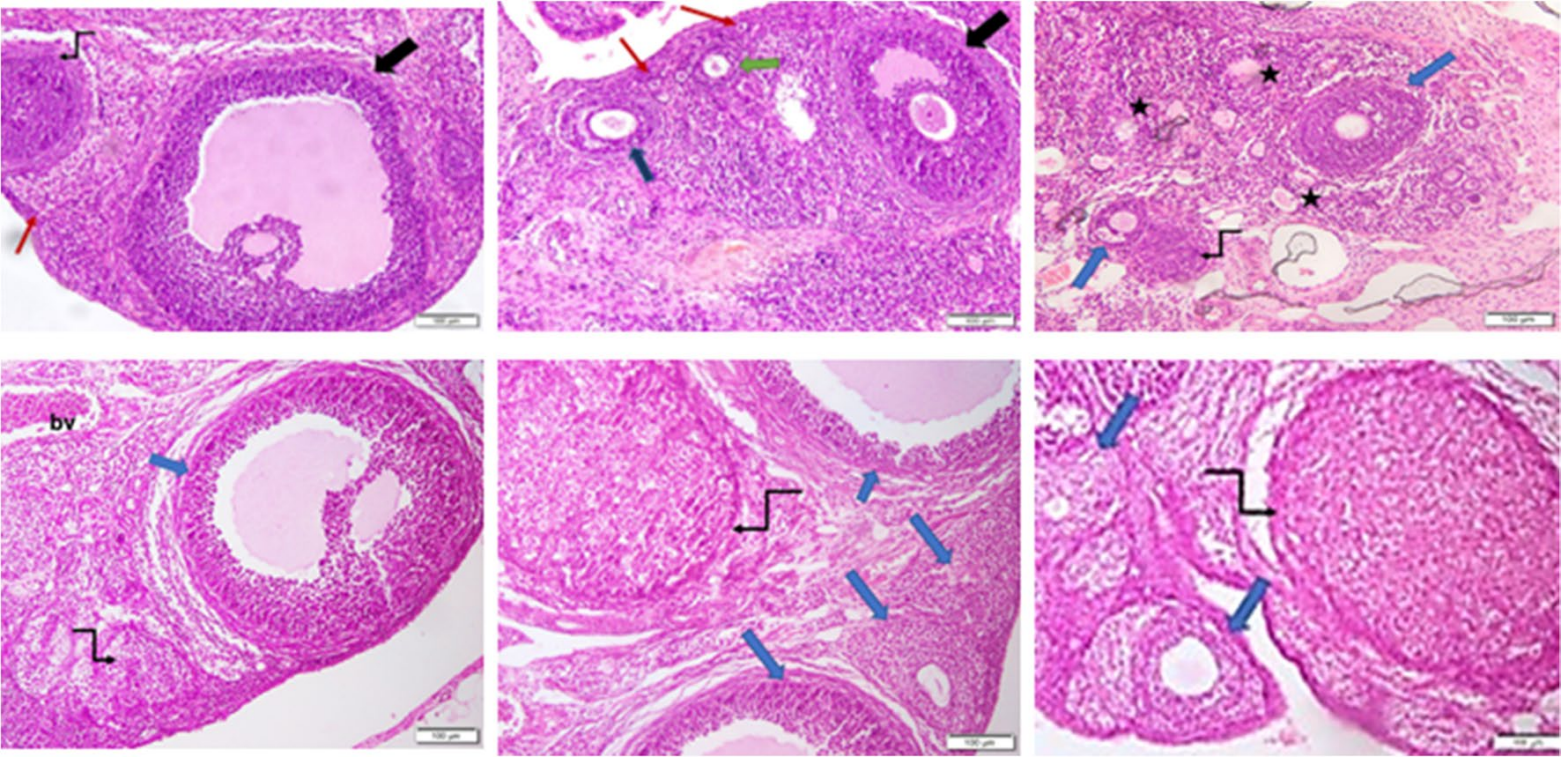
Photomicrographs of H&E-stained sections of ovaries of adult female *Wistar* albino rats. As ordered from left to right over two rows, **a.** Control group, exhibiting cortical stroma rich in primordial follicles (red arrow), mature graafian follicle (black arrow) as well as corpus luteum (kinked arrow) (× 400); **b.** AGO, showing cortical stroma rich in primordial follicles (red arrows), mature graafian follicle (black arrow), primary (green arrow) and secondary follicles with atretic oocyte (blue arrow) (× 400); **c.** QUET, showing, in addition to the corpus luteum (kinked arrow) and the secondary follicles with atretic oocyte (blue arrows), congested blood vessels (black stars) (× 400); **d.** CAFF, showing dilated congested blood vessels (bv), along with the corpus luteum (kinked arrow) and the secondary follicles with atretic oocyte (blue arrow) (× 400); **e.** AGO + CAFF wherein a large corpus luteum can be seen (kinked arrow) together with cystic follicles (blue arrows) (× 400) and **f.** QUET + CAFF with a large corpus luteum (kinked arrow) and follicles with atretic oocytes (blue arrows) (× 400). Adult female *Wistar* albino rats (N = 48) were equally subdivided into controls, AGO: 10 mg/kg agomelatine, oral, once daily, QUET: 10 mg/kg quetiapine, oral, once daily, CAFF: caffeine-containing beverages, as alternate-day coffee and cola, at room temperature, once daily, AGO + CAFF: caffeine-containing beverages followed by 10 mg/kg agomelatine, oral, once daily, QUET + CAFF: caffeine-containing beverages followed by 10 mg/kg quetiapine, oral, once daily. All administrations were adopted for 8 weeks

Both AGO and QUET monotherapies exhibited similar ovarian microstructures as the control, in terms of a cortical stroma rich in primordial follicles, along with mature Graafian follicles, primary oocytes, a co-existing corpus luteum, and both primary and secondary follicles and atretic oocytes **(**Figs. 12a-c**)**. Additionally, congested blood vessels were evident with QUET (Fig. 12c**)**. The ovaries of rats administered CAFF showed cystic follicles with atretic oocytes and dilated congested blood vessels **(**Fig. 12d**)**. The co-administration of AGO or QUET with CAFF yielded not only the persistence of cystic follicles with atretic oocytes but also the emergence of a large corpus luteum **(**Figs. 12e and f**)**.

### CAFF, alone or combined with AGO or QUET, reduced ovarian E2Rα

**Fig. 13:**
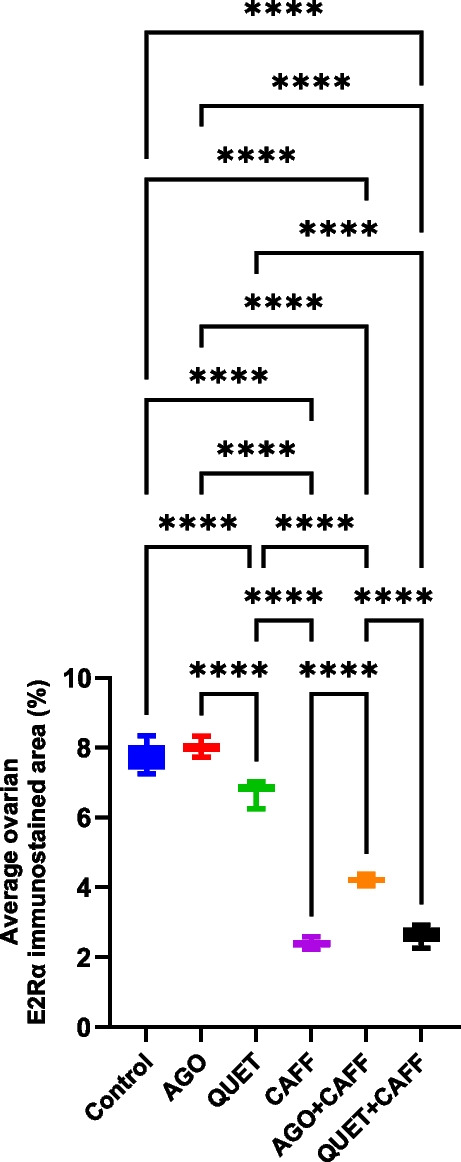
E2Rα immunoreactive area in rat ovaries (percent). Data are analyzed using Kruskal–Wallis, followed by post hoc Dunn’s test, and are represented as median and interquartile range. *ρ* < 0.05*; *ρ* < 0.01**; *ρ* < 0.001***; *ρ* < 0.0001****. Graphs are generated using Graph Pad Prism v.10.0.0. E2Rα: estrogen receptor alpha. Adult female *Wistar* albino rats (N = 48) were equally subdivided into controls, AGO: 10 mg/kg agomelatine, oral, once daily, QUET: 10 mg/kg quetiapine, oral, once daily, CAFF: caffeine-containing beverages, as alternate-day coffee and cola, at room temperature, once daily, AGO + CAFF: caffeine-containing beverages followed by 10 mg/kg agomelatine, oral, once daily, QUET + CAFF: caffeine-containing beverages followed by 10 mg/kg quetiapine, oral, once daily. All administrations were adopted for 8 weeks

**Fig. 14:**
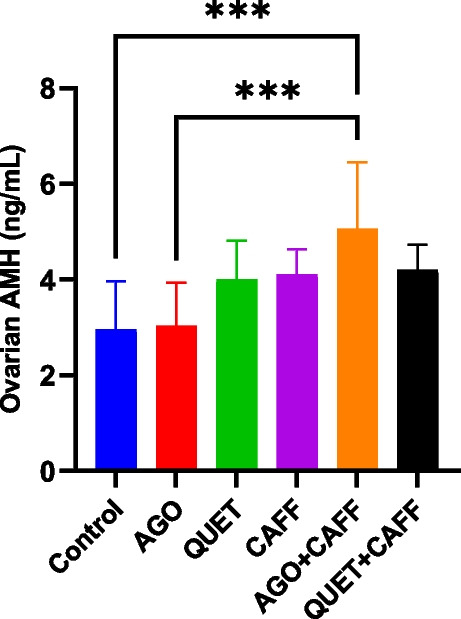
Ovarian AMH (ng/mL). Data are analyzed using ANOVA, followed by post hoc Tukey’s test, and are represented as mean ± standard deviation (SD). *ρ* < 0.05*; *ρ* < 0.001***. The graph is generated using Graph Pad Prism v.10.0.0. AMH: antimullerian hormone. Adult female *Wistar* albino rats (N = 48) were equally subdivided into controls, AGO: 10 mg/kg agomelatine, oral, once daily, QUET: 10 mg/kg quetiapine, oral, once daily, CAFF: caffeine-containing beverages, as alternate-day coffee and cola, at room temperature, once daily, AGO + CAFF: caffeine-containing beverages followed by 10 mg/kg agomelatine, oral, once daily, QUET + CAFF: caffeine-containing beverages followed by 10 mg/kg quetiapine, oral, once daily. All administrations were adopted for 8 weeks

As was with cortical E2Rα, CAFF significantly reduced ovarian E2Rα immunoreactive area relative to the control (*t*_(6)_ = 55.17, *ρ* < 0.0001) (Fig. 13 and Suppl. Figure 8). However, CAFF did not significantly alter either ovarian E2 or AMH level. In the context of ovarian E2Rα immunoreactive area, despite the significant increase following AGO + CAFF administration compared with CAFF (*t*_(6)_ = 55.17, *ρ* < 0.0001), it was persistently lower than the control (*t*_(6)_ = 55.17, *ρ* < 0.0001) (Fig. 13 and Suppl. Figure 8). Unlike brain AMH, AGO + CAFF significantly increased ovarian AMH level compared with the control (*F*_(5,42)_ = 6.009, *ρ* < 0.001) **(**Fig. 14**)**, which was not observed with either CAFF, AGO, or QUET + CAFF. Unlike AGO + CAFF, QUET + CAFF did not show a significant difference regarding the ovarian E2Rα immunoreactive area compared with CAFF, exhibiting a persistently reduced ovarian E2Rα immunoreactive area relative to the control (*t*_(6)_ = 55.17, *ρ* < 0.001) (Fig. 13 and Suppl. Figure 8). Similar to QUET + CAFF, QUET monotherapy was associated with a significantly lower ovarian E2Rα immunoreactive area compared with the control (*t*_(6)_ = 55.17, *ρ* < 0.0001) (Fig. 13 and Suppl. Figure 8). In contrast, when compared with the control, AGO monotherapy did not significantly alter the ovarian E2Rα-immunoreactive area. Relative to the control, neither AGO + CAFF nor QUET + CAFF significantly affected ovarian E2 level (Suppl. Figure 9). The correlations between ovarian E2, AMH, and E2Rα are illustrated in Suppl. Figure 10.

### QUET, more than AGO, antagonized CAFF-increased ovarian A2AR

**Fig. 15:**
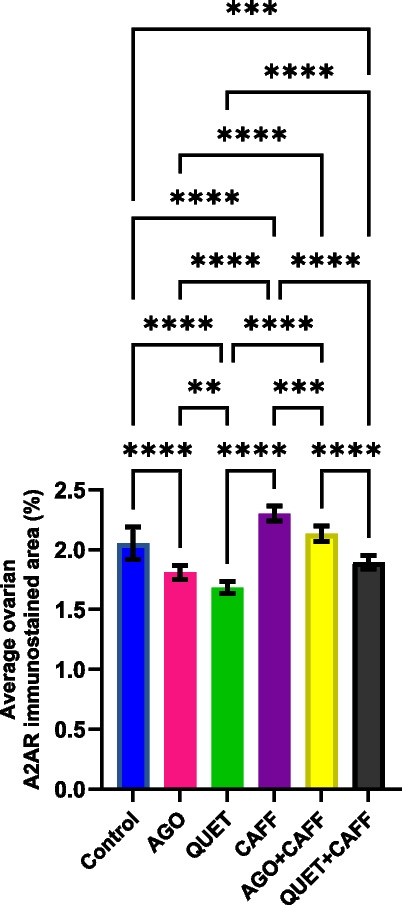
A2AR immunoreactive area in rat ovaries (percent). Data are analyzed using ANOVA, followed by post hoc Tukey’s test, and are represented as mean ± standard deviation. *ρ* < 0.05*; *ρ* < 0.01**; *ρ* < 0.001***; *ρ* < 0.0001****. Graphs are generated using Graph Pad Prism v.10.0.0. A2AR: adenosine receptor 2A. Adult female *Wistar* albino rats (N = 48) were equally subdivided into controls, AGO: 10 mg/kg agomelatine, oral, once daily, QUET: 10 mg/kg quetiapine, oral, once daily, CAFF: caffeine-containing beverages, as alternate-day coffee and cola, at room temperature, once daily, AGO + CAFF: caffeine-containing beverages followed by 10 mg/kg agomelatine, oral, once daily, QUET + CAFF: caffeine-containing beverages followed by 10 mg/kg quetiapine, oral, once daily. All administrations were adopted for 8 weeks

Relative to the control, CAFF significantly increased the ovarian A2AR-immunoreactive area (*F*_(5,54)_ = 86.72, *ρ* < 0.0001) (Fig. 15 and Suppl. Figure 11). Both AGO + CAFF and QUET + CAFF significantly reduced the ovarian A2AR-immunoreactive areas compared with CAFF (*F*_(5,54)_ = 86.72, *ρ* < 0.001, *ρ* < 0.0001, respectively), with QUET + CAFF having a more significant effect than AGO + CAFF (*F*_(5,54)_ = 86.72, *ρ* < 0.0001). When compared with the control, AGO + CAFF redeemed the ovarian A2AR-immunoreactive area, while QUET + CAFF significantly reduced it (*F*_(5,54)_ = 86.72, *ρ* < 0.001) (Fig. 15 and Suppl. Figure 11).

Compared with the control, both AGO and QUE monotherapies significantly reduced the ovarian A2AR-immunoreactive areas (*F*_(5,54)_ = 86.72, *ρ* < 0.0001); QUET had a more significant effect than AGO (*F*_(5,54)_ = 86.72, *ρ* < 0.01)*.* The reducing effects of AGO and QUET monotherapies significantly exceeded their respective combinations to CAFF (*F*_(5,54)_ = 86.72, *ρ* < 0.0001) (Fig. 15 and Suppl. Figure 11).

### AGO, more than QUET, synergized CAFF-increased ovarian MT2R

**Fig. 16:**
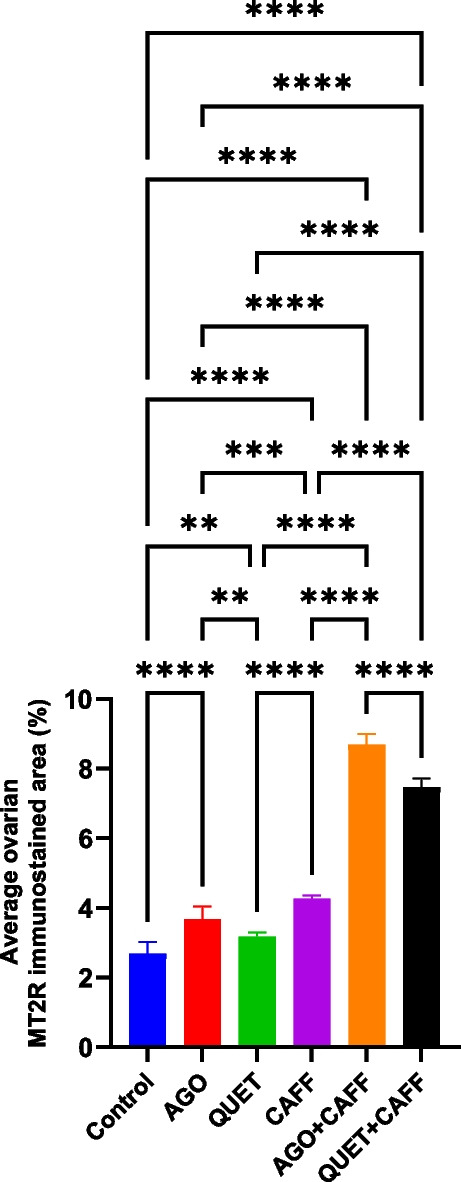
MT2R immunoreactive area of rat ovaries (percent). Data are analyzed using ANOVA, followed by post hoc Tukey’s test, and are represented as mean ± standard deviation. *ρ* < 0.05*; *ρ* < 0.01**; *ρ* < 0.001***; *ρ* < 0.0001****. Graphs are generated using Graph Pad Prism v.10.0.0. MT2R: melatonin receptor 2. Adult female *Wistar* albino rats (N = 48) were equally subdivided into controls, AGO: 10 mg/kg agomelatine, oral, once daily, QUET: 10 mg/kg quetiapine, oral, once daily, CAFF: caffeine-containing beverages, as alternate-day coffee and cola, at room temperature, once daily, AGO + CAFF: caffeine-containing beverages followed by 10 mg/kg agomelatine, oral, once daily, QUET + CAFF: caffeine-containing beverages followed by 10 mg/kg quetiapine, oral, once daily. All administrations were adopted for 8 weeks

Relative to the control, CAFF significantly increased the ovarian MT2R-immunoreactive area (*F*_(5,54)_ = 883.4, *ρ* < 0.0001) (Fig. 16 & Suppl. Figure 12). Both AGO and QUET synergized the CAFF effect by further increasing the ovarian MT2R-immunoreactive areas relative to CAFF (*F*_(5,54)_ = 883.4, *ρ* < 0.0001), exceeding that of the control (*F*_(5,54)_ = 883.4, *ρ* < 0.0001)*.* AGO + CAFF had a more significant effect than QUET + CAFF (*F*_(5,54)_ = 883.4, *ρ* < 0.0001) (Fig. 16 and Suppl. Figure 12).

Compared with the control, both AGO and QUET monotherapies significantly increased the ovarian MT2R-immunoreactive areas (*F*_(5,54)_ = 883.4, *ρ* < 0.0001;* ρ* < 0.01, respectively); AGO had a more significant effect than QUET (*F*_(5,54)_ = 883.4, *ρ* < 0.01). Such AGO and QUET effects were significantly lower than their respective effects when co-administered with CAFF (*F*_(5,54)_ = 883.4, *ρ* < 0.0001) (Fig. 16 and Suppl. Figure 12). Interestingly, unlike the lack of cortical correlation, ovarian MT2R was positively correlated to ovarian A2AR (*r* = *0.332, ρ* < 0.01).

### Ovarian MT2R was negatively correlated to ovarian E2Rα

**Fig. 17:**
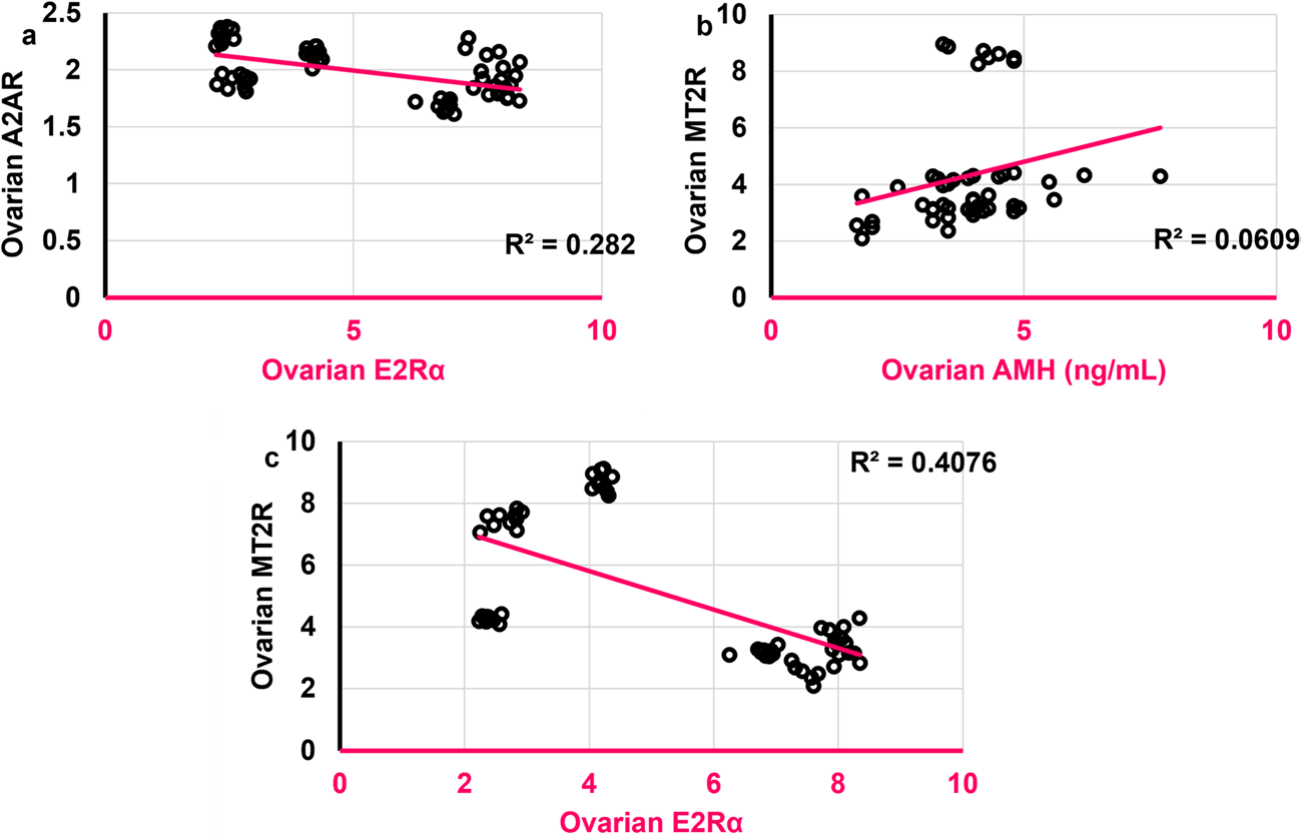
Scatter plots illustrating correlations between **a**. Ovarian E2Rα and ovarian A2AR and. **b.** Ovarian AMH (ng/mL) and ovarian MT2R and. **c.** Ovarian E2Rα and ovarian MT2R and. Scatter plots are generated using Microsoft Excel (Microsoft Office 365). Spearman *rho* correlation. Significant when *ρ* < 0.05. AMH: antimullerian hormone; E2Rα: estrogen receptor alpha; A2AR: adenosine receptor 2A; MT2R: melatonin receptor 2. Adult female *Wistar* albino rats (N = 48) were equally subdivided into controls, AGO: 10 mg/kg agomelatine, oral, once daily, QUET: 10 mg/kg quetiapine, oral, once daily, CAFF: caffeine-containing beverages, as alternate-day coffee and cola, at room temperature, once daily, AGO + CAFF: caffeine-containing beverages followed by 10 mg/kg agomelatine, oral, once daily, QUET + CAFF: caffeine-containing beverages followed by 10 mg/kg quetiapine, oral, once daily. All administrations were adopted for 8 weeks

Both ovarian E2 and E2Rα were negatively correlated to ovarian AMH (*r* = -0.314, *ρ* < 0.05; *r* = -0.469, *ρ* = 0.0008, respectively). As was with cortical A2AR, ovarian A2AR was negatively correlated to ovarian E2Rα (*r* = -0.484,* ρ* < 0.0001) **(**Fig. 17a**)**. Ovarian MT2R, but not A2AR, was positively correlated to ovarian AMH (*r* = 0.415, *ρ* = 0.003) **(**Fig. 17b**)**. Opposite to cortical hormones, ovarian MT2R was negatively correlated to ovarian E2Rα (*r* = **-0.594**,* ρ* < 0.0001) **(**Fig. 17c**)**; however, no significant correlation was found between either ovarian A2AR or MT2R and ovarian E2, unlike the positive correlation between cortical MT2R and brain E2.

### The maximum EEG peak and TTP were correlated to ovarian E2Rα and MT2R

**Fig. 18:**
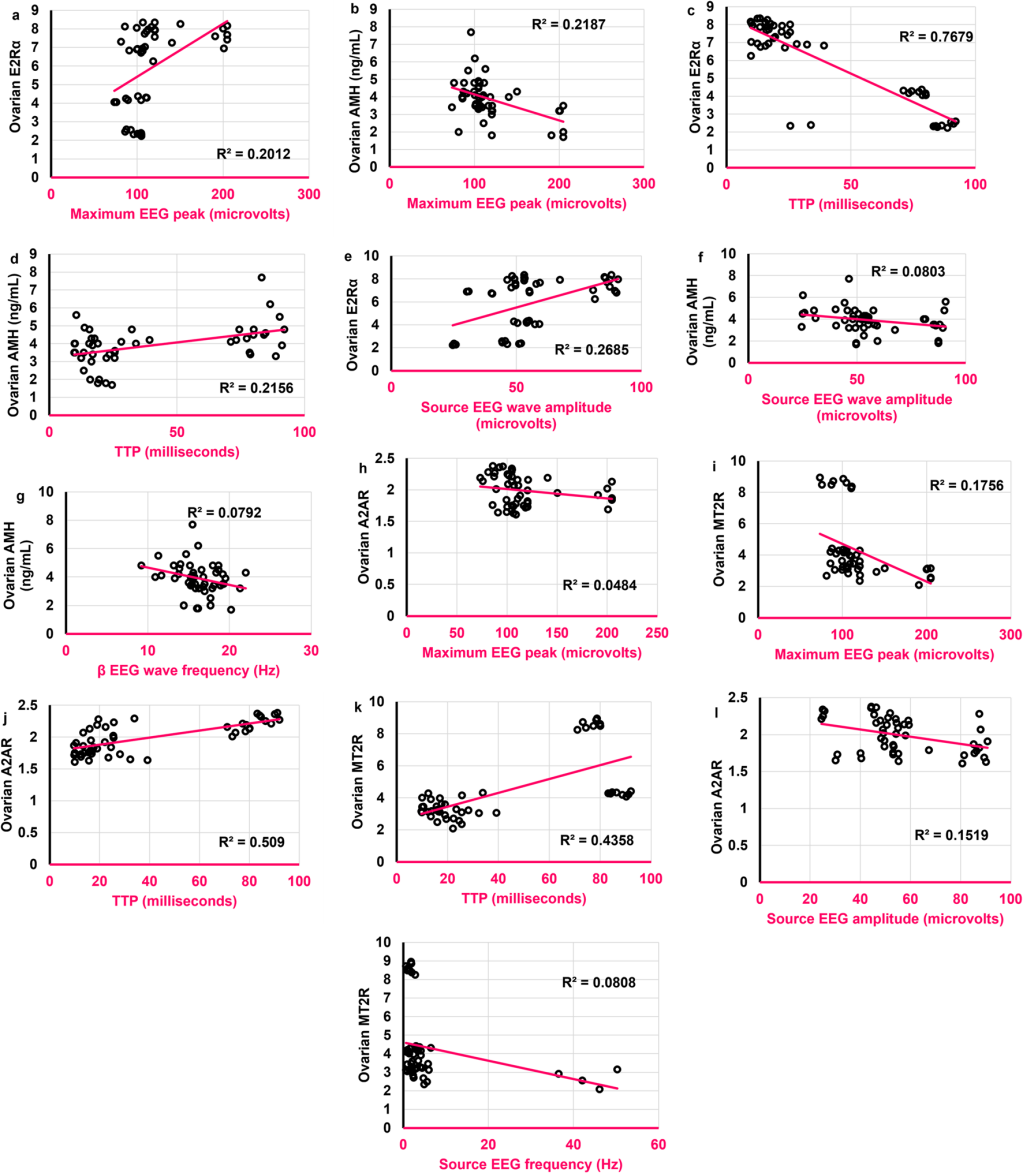
Scatter plots illustrating correlations between **a**. Maximum EEG peak (microvolts) and ovarian E2Rα. **b.** Maximum EEG peak (microvolts) and ovarian AMH (ng/mL). **c.** TTP (milliseconds) and ovarian E2Rα. **d.** TTP (milliseconds) and ovarian AMH (ng/mL). **e.** Source EEG amplitude (microvolts) and ovarian E2Rα. **f.** Source EEG amplitude (microvolts) and ovarian AMH (ng/mL). **g.** β EEG wave frequency (Hz) and ovarian AMH (ng/mL). **h.** Maximum EEG peak (microvolts) and ovarian A2AR. **i.** Maximum EEG peak (microvolts) and ovarian MT2R. **j.** TTP (milliseconds) and ovarian A2AR. **k.** TTP (milliseconds) and ovarian MT2R. **l.** Source EEG amplitude (microvolts) and ovarian A2AR. **m.** Source EEG frequency (Hz) and ovarian MT2R. Scatter plots are generated using Microsoft Excel (Microsoft Office 365). Spearman *rho* correlation. Significant when *ρ* < 0.05. TTP: Time-to-peak; E2: estradiol; AMH: antimullerian hormone; E2Rα: estrogen receptor alpha; A2AR: Adenosine receptor 2A; MT2R: Melatonin receptor 2. Adult female *Wistar* albino rats (N = 48) were equally subdivided into controls, AGO: 10 mg/kg agomelatine, oral, once daily, QUET: 10 mg/kg quetiapine, oral, once daily, CAFF: caffeine-containing beverages, as alternate-day coffee and cola, at room temperature, once daily, AGO + CAFF: caffeine-containing beverages followed by 10 mg/kg agomelatine, oral, once daily, QUET + CAFF: caffeine-containing beverages followed by 10 mg/kg quetiapine, oral, once daily. All administrations were adopted for 8 weeks

The maximum EEG peak was positively correlated to ovarian E2Rα (*r* = **0.535**,* ρ* < 0.001) **(**Fig. 18a**)** and negatively to ovarian AMH (*r* = -0.475,* ρ* = 0.001) **(**Fig. 18b**)**. TTP was negatively correlated to ovarian E2Rα (*r* = **-0.786**,* ρ* < 0.0001) **(**Fig. 18c**)** and positively to ovarian AMH (*r* = 0.366,* ρ* = 0.011) **(**Fig. 18d**)**. In contrast to TTP, the source EEG amplitude was positively correlated to ovarian E2Rα (*r* = 0.489,* ρ* = 0.0004) **(**Fig. 18e**)** and negatively to ovarian AMH (*r* = -0.320,* ρ* = 0.027) **(**Fig. 18f**)**. Similarly, β wave frequency was negatively correlated to ovarian AMH (*r* = -0.331,* ρ* = 0.021) **(**Fig. 18g**)**. None of the altered EEG aspects was significantly correlated to ovarian E2. Interestingly, the number of degenerated cortical cells was positively correlated to ovarian AMH (*r* = 0.450,* ρ* = 0.0013) and negatively to ovarian E2Rα (*r* = **-0.642**,* ρ* < 0.0001).

As for ovarian A2AR and MT2R, the maximum EEG peak was negatively correlated to both ovarian A2AR and MT2R (*r* = -0.317, *ρ* = 0.028;* r* = **-0.540**,* ρ* < 0.0001, respectively) **(**Figs. 18h and i**)**. In contrast, TTP was positively correlated to both ovarian A2AR and MT2R (*r* = **0.692**,* ρ* < 0.0001;* r* = **0.545**,* ρ* < 0.0001, respectively) **(**Figs. 18j and k**).** The source EEG wave amplitude was negatively correlated to ovarian A2AR (*r* = -0.386,* ρ* < 0.007) **(**Fig. 18l**)** but not significantly to ovarian MT2R. The source EEG frequency was negatively correlated to ovarian MT2R (*r* = -0.356,* ρ* = 0.013) **(**Fig. 18m**)** but not significantly to ovarian A2AR. Neither β nor δ wave was significantly correlated to either ovarian A2AR or MT2R. Moreover, the number of degenerated cortical cells was positively correlated to ovarian MT2R (*r* = 0.471,* ρ* = 0.0007) but not significantly to ovarian A2AR.

### Brain E2 was positively correlated to its ovarian analog

**Fig. 19:**
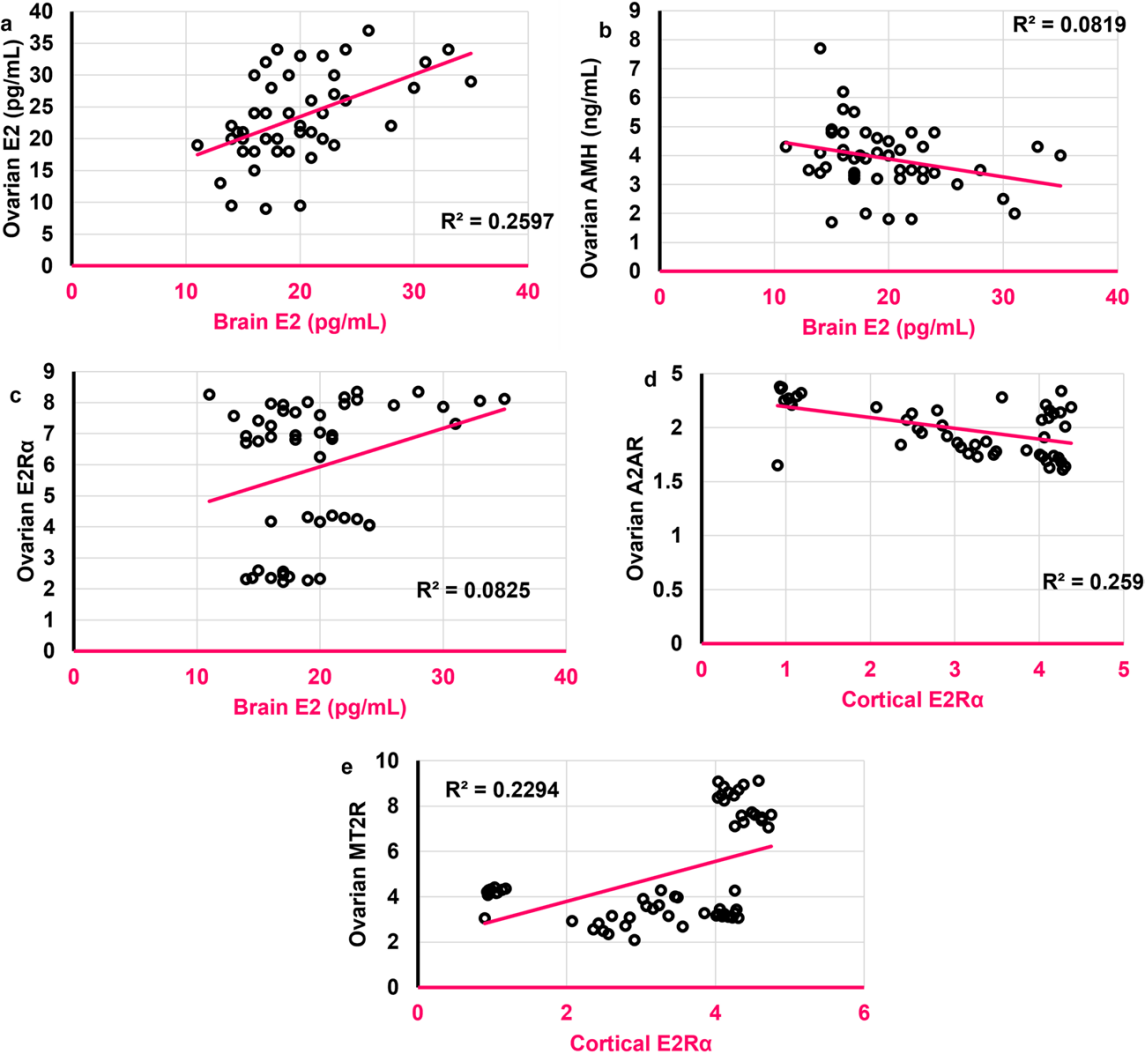
Scatter plots illustrating the correlations between **a**. Brain E2 (pg/mL) and ovarian E2 (pg/mL). **b.** Brain E2 (pg/mL) and ovarian AMH (ng/mL). **c.** Brain E2 (pg/mL) and ovarian E2Rα. **d.** Cortical E2Rα and ovarian A2AR. **e.** Cortical E2Rα and ovarian MT2R. Scatter plots are generated using Microsoft Excel (Microsoft Office 365). Spearman *rho* correlation. Significant when *ρ* < 0.05. E2: estradiol; AMH: antimullerian hormone; E2Rα: estrogen receptor alpha; A2AR: Adenosine receptor 2A; MT2R: Melatonin receptor 2. Adult female *Wistar* albino rats (N = 48) were equally subdivided into controls, AGO-treated: 10 mg/kg agomelatine, oral, once daily, QUET-treated: 10 mg/kg quetiapine, oral, once daily, CAFF: caffeine-containing beverages, as alternate-day coffee and cola, at room temperature, once daily, AGO + CAFF: caffeine-containing beverages followed by 10 mg/kg agomelatine, oral, once daily, QUET + CAFF: caffeine-containing beverages followed by 10 mg/kg quetiapine, oral, once daily. All administrations were given for 8 weeks

Brain E2 was positively correlated to both ovarian E2 (*r* = **0.509**,* ρ* < 0.001) and ovarian E2Rα (*r* = 0.311,* ρ* = 0.032) **(**Figs. 19a and c**)** and negatively to ovarian AMH (*r* = -0.285,* ρ* = 0.049) **(**Fig. 19b**)**. Cortical E2Rα was negatively correlated to ovarian A2AR (*r* = -0.337,* ρ* = 0.009) **(**Fig. 19d**)** and positively to ovarian MT2R (*r* = 0.459,* ρ* = 0.0003) **(**Fig. 19e**)**. However, no significant correlation was found between brain E2 and either ovarian A2AR or MT2R. Cortical E2Rα was neither correlated to ovarian AMH nor to ovarian E2Rα.

No significant correlation was found between brain AMH and either ovarian E2, AMH, E2Rα, A2AR, or MT2R.

### Cortical A2AR was negatively correlated to ovarian E2Rα

**Fig. 20:**
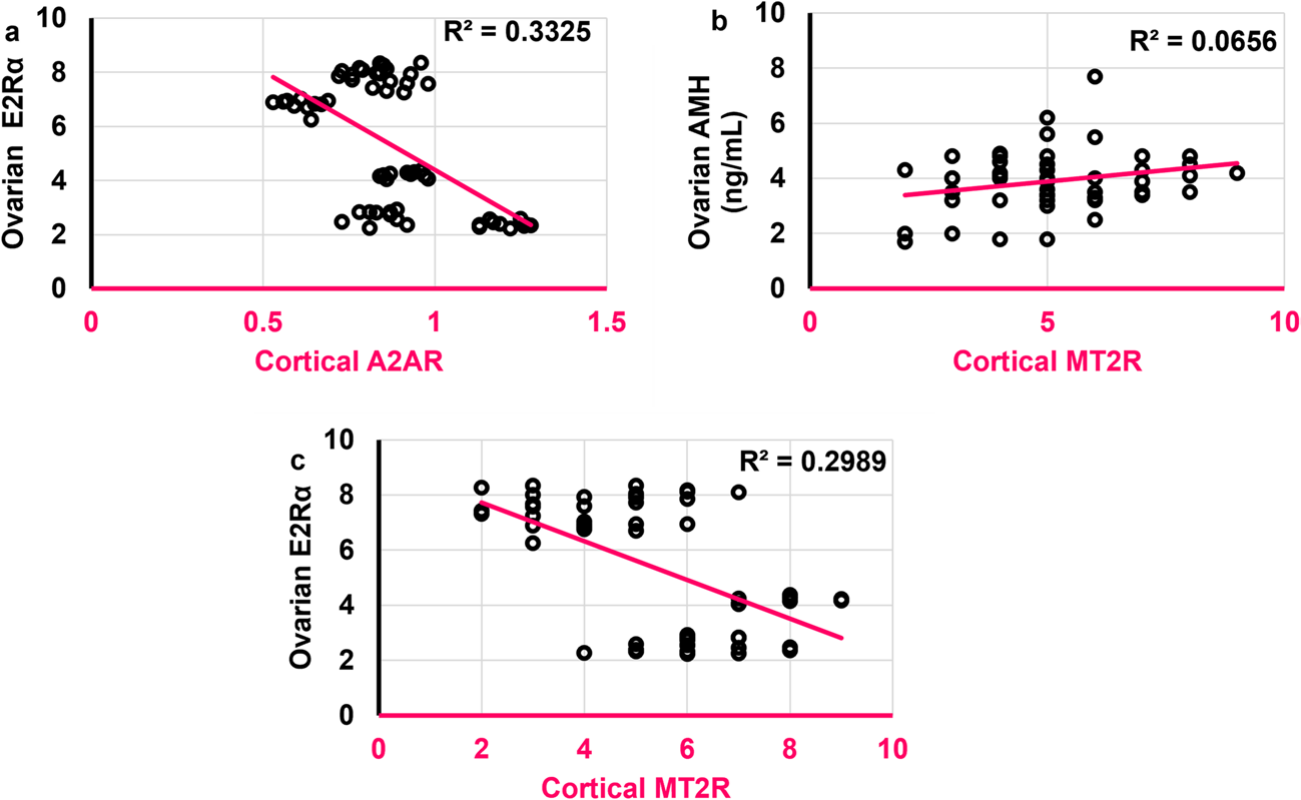
Scatter plots illustrating correlations between **a**. Cortical A2AR and ovarian E2Rα. **b.** Cortical MT2R and ovarian AMH (ng/mL). **c.** Cortical MT2R and ovarian E2Rα. Scatter plots are generated using Microsoft Excel (Microsoft Office 365). Spearman *rho* correlation. Significant when *ρ* < 0.05. AMH: antimullerian hormone; E2Rα: estrogen receptor alpha; A2AR: adenosine receptor 2A; MT2R: melatonin receptor 2. Adult female *Wistar* albino rats (N = 48) were equally subdivided into controls, AGO: 10 mg/kg agomelatine, oral, once daily, QUET: 10 mg/kg quetiapine, oral, once daily, CAFF: caffeine-containing beverages, as alternate-day coffee and cola, at room temperature, once daily, AGO + CAFF: caffeine-containing beverages followed by 10 mg/kg agomelatine, oral, once daily, QUET + CAFF: caffeine-containing beverages followed by 10 mg/kg quetiapine, oral, once daily. All administrations were adopted for 8 weeks

Cortical A2AR was negatively correlated to ovarian E2Rα (*r* = -0.463,* ρ* = 0.0002) **(**Fig. 20a**)**, while cortical MT2R was positively correlated to ovarian AMH (*r* = 0.374,* ρ* = 0.009) **(**Fig. 20b**)** and negatively to ovarian E2Rα (*r* = -0.444,* ρ* = 0.0004) **(**Fig. 20c**)**. No significant correlation was found between cortical A2AR and either ovarian E2 or ovarian AMH. As was with cortical A2AR, no significant correlation was detected between cortical MT2R and ovarian E2.

### Cortical A2AR and MT2R were strongly and positively correlated to their respective ovarian analogs

**Fig. 21:**
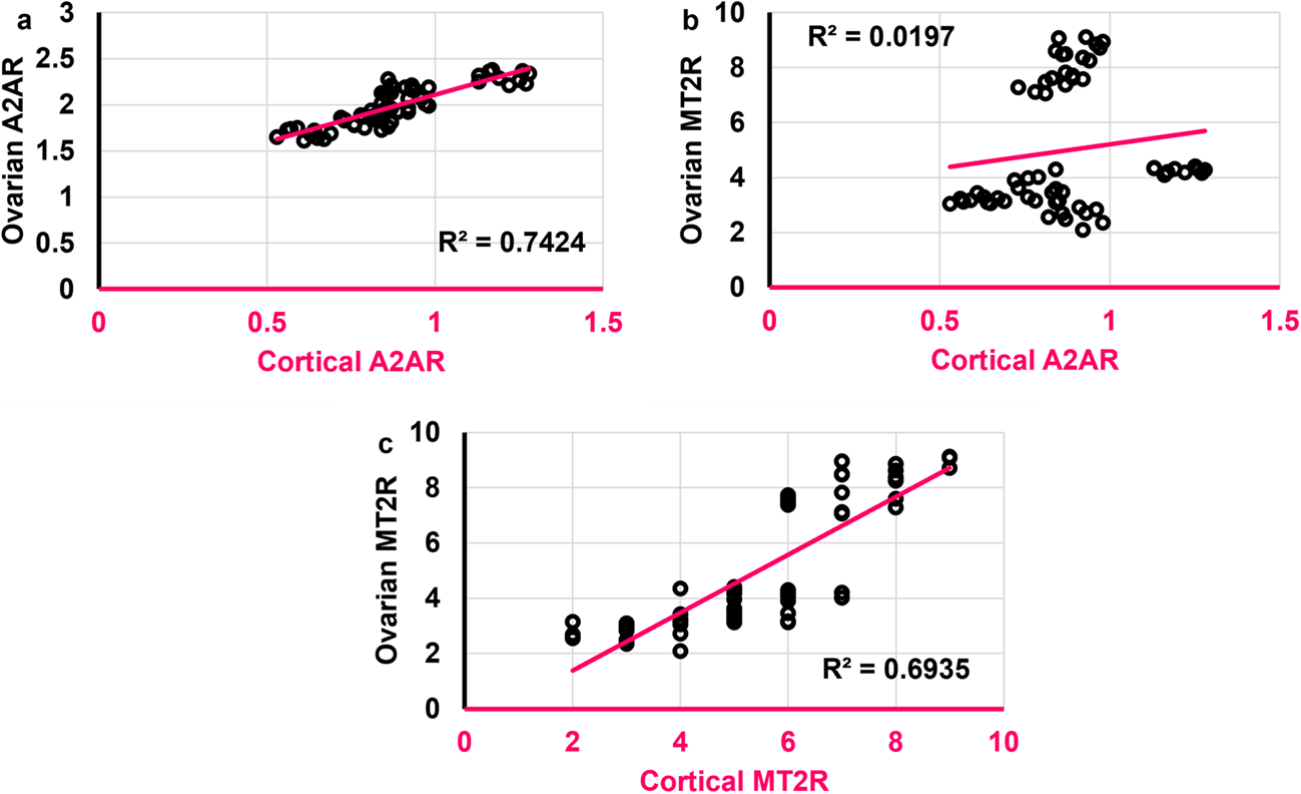
Scatter plots illustrating correlations between **a**. Cortical A2AR and ovarian A2AR. **b.** Cortical A2AR and ovarian MT2R. **c.** Cortical MT2R and ovarian MT2R. Scatter plots are generated using Microsoft Excel (Microsoft Office 365). Spearman *rho* correlation. Significant when *ρ* < 0.05. A2AR: adenosine receptor 2A; MT2R: melatonin receptor 2. Adult female *Wistar* albino rats (N = 48) were equally subdivided into controls, AGO: 10 mg/kg agomelatine, oral, once daily, QUET: 10 mg/kg quetiapine, oral, once daily, CAFF: caffeine-containing beverages, as alternate-day coffee and cola, at room temperature, once daily, AGO + CAFF: caffeine-containing beverages followed by 10 mg/kg agomelatine, oral, once daily, QUET + CAFF: caffeine-containing beverages followed by 10 mg/kg quetiapine, oral, once daily. All administrations were adopted for 8 weeks

Cortical A2AR was positively correlated to both ovarian A2AR and ovarian MT2R (*r* = **0.874**,* ρ* < 0.0001;* r* = 0.334,* ρ* = 0.009, respectively) **(**Figs. 21a and b**)**. Cortical MT2R was positively correlated to ovarian MT2R (*r* = **0.857**,* ρ* < 0.0001) **(**Fig. 21c**)** but not significantly to ovarian A2AR.

Suppl. table 1 summarizes the findings.

## Discussion

To the best of our knowledge, this is the first study to compare the effects of CAFF to those of CAFF combined with AGO or QUET, on EEG, cortical microstructure, on one hand, and estrus cycle progression as well as ovarian microstructure, on the other hand. We explored the potential involvement of cortical and ovarian A2AR and MT2R and addressed the brain-ovarian crosstalk by assessing potential links between EEG, microstructural changes, and central as well as ovarian endocrinal milieu. Our comparison included the variations in brain and ovarian E2, AMH, and E2Rα.

In this study, a reduced maximum EEG peak as well as cortical neurodegeneration was observed with CAFF and persisted following the co-administration of AGO and QUET. Such reduced maximum EEG peak was previously reported in the presence of cortical atrophy when recording using scalp electrodes (He et al. 2021). Such association was corroborated herein by detecting a negative correlation between the number of degenerated cortical cells and maximum EEG peak. Reduced maximum EEG peak has been reported in patients with AD and elevated beta-amyloid, even in those with apparently normal cognition, and was correlated to hyperexcitability, preceding a hypo-excitability state (Devos et al. 2022). EEG is a potential marker of neurodegeneration, as indicated herein. In our study, the associated CAFF-reduced cortical E2Rα immunoreactivity suggested a possible defective cognition, given the role of estrogen signaling in such neurological function, consistent with a previous study showing the association between low estrogen level and impaired cognition (Au et al. 2016). The role of defective estrogen signaling in neurodegeneration and impaired cognition has been reported (Inestrosa et al. 1998; Woolley 1999) and was recently corroborated in ovariectomized rats (Fang et al. 2018). The concurrent slowed β frequency highlighted the potential of reduced cognition with chronic CAFF consumption considering the link between beta activity and cognition (Miller et al. 2018).

Moreover, the enhanced cortical A2AR with CAFF supported the likelihood of impaired cognition since the inactivation of A2AR possibly improves cognition (Blum et al. 2018). A remote possibility would be cognitive enhancement based on a previous study demonstrating the boosting activity of adenosine receptors activation in cognition (Chen 2014).

While most studies relied on the power of EEG waves, few have investigated the significance of alterations of global EEG frequency as relevant to cognition and hyperexcitability states, added to the lack of studies tracking the variations of maximum EEG peak. In this study, especially in the presence of reduced cortical E2Rα with CAFF, a reduced maximum EEG peak could reflect a lower propensity for epileptogenesis, given the positive link between estrogens and epileptogenesis (Velíšková et al. 2010). Such reduction of epileptogenesis encountered with chronic CAFF administration was supported by a previous meta-analysis and systematic review suggesting that chronic CAFF consumption in animal models protects against seizures (van Koert et al. 2018). Currently, no consensus has been reached concerning the link between CAFF and epilepsy in humans (Bauer & Sander 2019; van Koert et al. 2018). Based on existing literature focusing on spectral EEG analysis, rather than global EEG changes, no conclusive evidence of CAFF link to epileptogenesis has been reported. It is difficult to establish a definite causal relationship between CAFF and seizure risk, attributed, in part, to discrepancies between animal studies (Bauer & Sander 2019). Nonetheless, given the observed EEG and central endocrinal variations herein, CAFF could exert an anti-epileptogenic effect.

Unlike recent studies identifying CAFF as a neuroprotective factor in disease models (Pereira-Figueiredo et al. 2021; Ruggiero et al. 2022; Schepici et al. 2020), only a few reports outlined the differential effects of low versus medium-to-high CAFF consumption on normal subjects. For instance, (Oboh et al. 2017) have suggested that 50–100 mg/kg CAFF consumption in normal rats can reduce the effects of donepezil, an anti-Alzheimer’s medication; however, the authors provided no justification of their findings. Our study indicated that administration of low dose CAFF, but for a prolonged duration, triggered neuropathologic features. Apart from the reduced cortical E2Rα, CAFF-associated neurodegeneration could be justified in terms of increased cortical A2AR previously observed with prolonged CAFF intake (Snel & Lorist 2011). The implication of A2AR in neurodegenerative disorders has been reported (Augusto et al. 2013; Pedata et al. 2017). Complementing the existing theory on the neuroprotective effects of CAFF as attributed to A2AR antagonism, with the subsequent promotion of dopaminergic neurotransmission (Watanabe & Uramoto 1986), prolonged CAFF administration could breech such theory by inducing an adaptive upregulation of A2AR, as reported previously following 2-week daily 400 mg CAFF consumption (Varani et al. 1999). The notion that the enhancing activity of CAFF over cognition is attributed to A2AR antagonism can explain the cognitive deterioration following chronic CAFF consumption, owing to a compensatory increase in cortical A2AR (Faivre et al. 2018; Temido-Ferreira et al. 2020). Opposing the anti-epileptogenic activity of CAFF, increased A2AR has been linked to a higher risk of epileptogenesis and related neuronal damage (Augusto et al. 2021).

The slowing of fast EEG waves encountered with CAFF and QUET– slowed β in the case of CAFF and slowed γ in the case of QUET– culminated in significant slowing of global source EEG wave, attaining a δ wave range when combining CAFF with QUET. The notion that source EEG frequency is negatively correlated to cortical E2Rα, the latter attaining its highest immunoreactivity with QUET + CAFF, justified such prominent EEG slowing.

Adopting the increased cortical E2Rα immunoreactivity observed with QUET + CAFF as a neuro-reparative attempt to preserve some neuronal survival and integrity (Jäkel & Dimou 2017) can justify the claimed link between slow EEG activity and enhanced neuronal communication as that observed between the prefrontal cortex and other brain areas in a previous rodent study (Mofleh & Kocsis 2021). Against such adopted hypothesis is the persistent cortical neurodegeneration featured with QUET + CAFF, especially in the presence of multinucleated cells, known to occur with chronic inflammatory states (Hazra et al. 2023). Furthermore, a link between estrogen receptors and the higher prevalence and more aggressive pathologic features of neurodegenerative disorders in middle-aged females compared with males has been reported (Association 2019; Barnes et al. 2005). Nonetheless, the slowing of source EEG wave remains a favorable sign indicating a presumptive cognitive enhancement based on the increased cortical MT2R immunoreactivity observed with QUET + CAFF and the negative correlation between source EEG frequency and cortical MT2R, given the contribution of MT2R to better cognition (Xu et al. 2015). The negative correlation between source EEG frequency and cortical E2Rα corroborated such cognitive amelioration with QUET + CAFF, relying on the relevance of estrogen to cognitive functions (Hwang et al. 2020; Russell et al. 2019). Perhaps neuronal repair was too slow to manifest; the associated increased brain E2 level could support neuronal repair, though, consistent with the neuroprotective and anti-inflammatory roles of E2, along with its ability to improve neurogenesis (Yanguas-Casás et al. 2019). Another EEG aspect noticed with QUET + CAFF was the emergence of a reduced δ amplitude. δ amplitude was negatively correlated to cortical E2Rα, justifying such occurrence, which was missed in both CAFF –when cortical E2Rα was reduced– and QUET monotherapies, exhibiting a much less increase of cortical E2Rα relative to QUET + CAFF. The reduced δ amplitude is considered a favorable sign in the context of cognition (Roascio et al. 2022; Roohi-Azizi et al. 2017). EEG findings and alterations in the central endocrinal milieu suggested QUET + CAFF as a potential cognitive enhancer.

Fortunately, the persistent neurodegeneration with AGO + CAFF did not hinder the potential antiseizure activity, manifested as delayed TTP, opposite to the proconvulsant effect of accelerated EEG peaks when modifying the times of sleep (Karoly et al. 2021). In the context of epileptogenesis, a retrospective analysis of continuous EEG recordings obtained from adult male and female patients with focal epilepsy revealed the occurrence of seizures near the EEG peak (Leguia et al. 2021). Furthermore, the positive correlation between TTP and cortical A2AR, redeemed herein, was consistent with such antiseizure activity, given the implication of A2AR in seizures as previously observed in young rats when the activation of central A2AR lowered the threshold for hyperthermia-induced seizures (Fukuda et al. 2011). While adenosine itself, by acting on A1R, was recently adopted as an endogenous anticonvulsant neurotransmitter, the activation of A2AR triggered convulsions (Baltos et al. 2023). Perhaps reduced neuroinflammation, as could be deduced from the absence of inflammatory multinucleated cells, could have contributed. Persistent neurodegeneration seen with AGO + CAFF did not occur when AGO (10 mg/kg) was administered, especially in the presence of elevated brain E2. This is consistent with a previous study in which AGO was administered at a higher dose (40 mg/kg) to a kainic acid-induced model of status epilepticus in rats, and the authors indicated that the anticonvulsant effect of AGO could occur, even if AGO did not exert a neuroprotective activity (Tchekalarova et al. 2019). Although, with prolonged AGO monotherapy, no overt EEG changes denoted a better cognitive state than controls, the increased brain E2, together with enhanced cortical E2Rα immunoreactivity, associated with a similar cortical microstructure as the control, support previous studies highlighting the cognitive enhancing activity of both estrogenic signaling (Boyle et al. 2021) and AGO (Su et al. 2023). The role of estrogen in AGO-mediated cognitive amelioration has been demonstrated in ovariectomized rats (El-Khatib et al. 2020). However, the lack of EEG epileptogenic activity and the concurrently reduced cortical A2AR immunoreactivity emphasize the potential safety of AGO concerning epileptogenesis as previously reported in a mouse model of epilepsy (Dastgheib & Moezi 2014) and patients (Jiang et al. 2024). The associated increased δ frequency, still within δ range, was also recovered in ‘quiet’ wakefulness in humans (Sachdev et al. 2015), mimicking the recording setting herein. Furthermore, reduced δ slowing is consistent with the anti-epileptogenic potential of AGO + CAFF (Jalilifar et al. 2016). In the presence of neurodegeneration, cognitive recovery might not be complete, as indicated by reduced δ activity, especially in the context of memory, as occurred with agents affecting NREM-affecting (Uygun & Basheer 2022).

Notably, unlike the normal EEG tracings with prolonged AGO treatment, the increased source EEG amplitude and slowing of γ wave provoked by prolonged QUET monotherapy were consistent with the sleep-promoting properties of QUET. γ wave slowing was previously observed with older hypnotics, such as barbiturates, and corresponded to slow behavioral reactions when recording frontal cortex EEG in young rats (Insel et al. 2012). The relative safety of QUET as regards epileptogenesis and the concurrent reduction of cortical A2AR immunoreactivity are consistent with a previous study in young rats indicating that activation of central A2AR lowered the threshold for hyperthermia-induced seizures (Fukuda et al. 2011). As QUET showed the lowest cortical A2AR immunoreactivity, our findings suggested a link between cortical A2AR and such EEG changes by identifying the correlations between cortical A2AR, on one side, and source EEG amplitude and γ wave frequency, on the other side, negative for the former but positive for the latter. Paradoxically, the increased source EEG amplitude denoting a pro-convulsant activity, as previously suggested by an amplitude-integrated EEG recorded in children (Glass et al. 2013), outlined the intricated role of adenosinergic signaling in epileptogenesis and suggested that receptors, other than A2AR, paly a chief role. Therefore, although bearing a low risk of epileptogenesis, QUET could still precipitate seizures (Agrawal & Mula 2019).

From an ovarian aspect, CAFF was associated with cystic follicles and atretic oocytes, as well as congested blood vessels. Such ovarian microstructural derangement was not associated with significantly delayed estrus cycle progression, involving mainly proestrus (follicular) and estrus (ovulation) phases, suggesting defective estrogenic signaling possibly from reduced ovarian E2Rα immunoreactivity, yet with unaltered ovarian E2 and AMH levels. The presence of cystic follicles with atretic oocytes and the more prolonged proestrus and estrus phases could denote anovulatory cycles. The findings of (Broderick & Malave 2014) agreed, in part, with our results showing that CAFF did not modify estrogen levels. Still, the significance of reduced E2Rα in fertility was identified when E2Rα-knocked out mice were infertile, despite intact central feedback mechanisms increasing plasma E2 levels (Couse et al. 2003); whereas mice with defective E2Rβ were fertile (Krege et al. 1998). E2Rα are activated in an estrogen-independent manner (Smith 1998) to exert rapid non-genomic actions (Kelly & Levin 2001), such as preovulatory follicles maturation and ovulation (Gérard & Robin 2019). The lack of sound delay in estrus cycle progression in our CAFF model supports, in part, previous reports arguing against a link between CAFF intake and female infertility (Bu et al. 2020; Hakim et al. 1998).

A previous study reported infertility in females consuming ≥ 3 cups of coffee at 100 mg caffeine/cup daily (Stanton & Gray 1995); this was significantly higher than the amount used in this study. Extrapolating the human daily allowed amount of CAFF of 100 mg to rat dose yields 1.43 mg/kg, higher than the daily doses used in our study (1.32 and 0.72 mg/kg), explaining the lack of significant effects over estrus cycle progression and female ovarian E2 and AMH in our model. A larger dose than that used in this study was deemed safe by the European Food Safety Authority (EFSA) (2015) and US Food and Drug Administration (2018), allowing a daily consumption of up to 400 mg CAFF in healthy adults and 200 mg in pregnant and lactating women. Some studies investigating CAFF and female fertility were previously discussed by (Cao et al. 2016), revealing that no consensus has been reached on this matter.

Notably, CAFF enhanced the ovarian immunoreactivity of both A2AR and MT2R. Previously, the consumption of two cups of coffee at night by healthy individuals increased serum melatonin levels owing to a competition between CAFF and melatonin for the same metabolizing enzyme (Ursing et al. 2003). Enhanced melatonin signaling has an antioxidant effect that protects granulosa cells and oocytes against oxidative damage (Tamura et al. 2008; Tanabe et al. 2015). This could not be verified in our model given the cystic and atretic ovarian follicles, possibly attributed to low ovarian E2Rα (Fernando & Rombauts 2014). Unlike previous studies on the role of melatonin in the regulation of female fertility, limited data concerning adenosine are available, mostly discussing adenosine as a target for arresting ovarian cancer cell proliferation being increased in the tumor milieu (Allard et al. 2020; Sureechatchaiyan et al. 2018). One study reported increased brain cyclic adenosine monophosphate, an adenosine derivative, activating downstream signaling pathways to trigger ovulation (Land et al. 2022). The ovarian MT2R-E2Rα negative correlation supported the sequential role of melatonergic/estrogenic signaling in terms of menstrual (estrus) cycle progression. As enhanced melatonergic signaling is required for the follicular (proestrus) phase, priming estrogen signaling is required for ovulation to proceed (Olcese 2020; Tamura et al. 2009), so that, in the presence of reduced ovarian E2Rα versus increased ovarian MT2R, a delayed progression to the estrus phase is anticipated. The additional positive correlation between ovarian A2AR and MT2R might have culminated in arrest in the estrus phase and a delayed progression to the secretory phase. Conversely, the favorable effects of caffeine on estrus cycle disturbances have been demonstrated in an acute cocaine addiction model with the implication of central neuroprotective mechanisms (Broderick & Malave 2014).

Combining CAFF with AGO or QUET had no effects on estrus cycle progression. Remarkably, differential phase affection in combination regimens mainly involved metestrus (early secretory) and diestrus (late secretory) phases. A similar ovarian microscopic appearance as CAFF was evident, which was accompanied by a large corpus luteum. The presence of a large corpus luteum corresponds to a high progesterone level (vom Saal 1994), suppressing estrogen as previously shown (Bondi et al. 2014) and detected herein by a persistently low ovarian E2Rα. Additionally, when CAFF was consumed with AGO or QUET, unlike CAFF alone, ovarian A2AR immunoreactivity was redeemed or reduced compared with the control, respectively. In contrast, ovarian MT2R immunoreactivity was further enhanced. Such similarities and discrepancies between CAFF and combinations suggest the involvement of adenosinergic and melatonergic signaling and a cyclical pattern of release.

Apart from two studies, one reporting the implication of cyclic adenosine monophosphate in progesterone synthesis (Roy et al. 2009) and the other highlighting the role of another adenosine derivative, adenosine triphosphate, in ovulation, the blockade of which receptor prevented ovulation in normal rats (Inoue et al. 2023), there are no studies justifying the arrest in secretory phases, especially in presence of reduced ovarian A2AR with QUET + CAFF. Such delay was replicated with AGO + CAFF despite the redemption of ovarian A2AR to the control level. In such a case, the concomitantly increased ovarian AMH, in the presence of persistently reduced ovarian E2Rα immunoreactivity, against increased ovarian MT2R immunoreactivity, exceeding QUET + CAFF, could have yielded such changes. Additionally, ovarian MT2R was positively correlated to ovarian AMH. The disturbed ovarian microstructure, despite the absence of significant estrus cycle delay, suggested the adversity of ovarian AMH increase. The potential implication of AMH as a marker for ovarian disturbances, as was with polycystic ovary syndrome, especially when the latter is associated with anovulatory cycles (Barbotin et al. 2019; Pasquali 2018), supports such interpretation.

The novelty of our study lies in determining the linkage between brain and ovarian changes, which provides insights into systemic endocrinal-based neurotherapeutics. the maximum EEG peak was positively correlated to ovarian E2Rα, addressing the brain-ovarian crosstalk. This was emphasized by observing their concurrent reductions with CAFF, both are aspects of impaired cognition. Such reductions were persistent when CAFF was combined with AGO or QUET, despite enhanced cortical E2Rα. Conformant to the neuroprotective role of estrogen (Bustamante-Barrientos et al. 2021; Simpkins et al. 2012), our study revealed that the number of degenerated cortical cells was negatively correlated to ovarian E2Rα. Such findings supported the implication of systemic estrogen in brain functions; therefore, hormonal replacement therapy could be employed for the treatment of some neurodegenerative disorders (Boyle et al. 2021; Brann et al. 2007; Russell et al. 2019). Given the pro-convulsant activity of E2 (Frank & Tyson 2020), the positive correlation between brain E2 and ovarian E2Rα detected herein highlights the anti-epileptogenic activity of CAFF, alone or combined with AGO or QUET, owing to reduced ovarian E2Rα. The implication of reduced melatonin signaling and hyperexcitability states could verify such observation, corroborated by the negative correlation between the maximum EEG peak and ovarian MT2R. Our findings are in line with those of previous studies and systematic reviews adopting melatonin as an adjuvant seizure controller based on its anticonvulsant potentiality (Khan et al. 2021; Liu et al. 2024; Maghbooli et al. 2023). Specific targets of MT2R remain to be determined.

TTP, negatively correlated to ovarian E2R and positively correlated to ovarian MT2R, offers another EEG indicator of epileptogenesis. A delayed TTP, together with reduced ovarian E2Rα, against increased ovarian MT2R, is a favorable marker in epileptogenesis. The implication of reduced melatonin signaling and hyperexcitability states could also be applied, consistent with the anticonvulsant potentiality of melatonin (Khan et al. 2021; Liu et al. 2024; Maghbooli et al. 2023). Moreover, the positive link between TTP and ovarian AMH was consistent with the increase in AMH in females during the seizure-free period compared with the control and those during the seizure episodes (Harden et al. 2016). The notion that a positive link existed between TTP and the number of degenerated cortical cells as well as both cortical and ovarian A2AR supported a recent study reporting the contribution of A2AR in seizure-induced neurodegeneration in a rat model of epilepsy (Xu et al. 2022). TTP has been suggested to provide insights into sleep behavior (Stokes & Prerau 2020).

Given that A2AR could be related to either the arousal effect of CAFF (Huang et al. 2005), or a rapid recovery following sleep deprivation (Sheth et al. 2014), a delayed TTP might indicate a rapid recovery from sleep deprivation based on a significantly lower ovarian A2AR encountered with AGO + CAFF and QUET + CAFF when compared with CAFF.

Apart from the positive correlations to cortical A2AR, neither maximum EEG peak nor TTP was significantly correlated to either cortical, E2, E2Rα, or MT2R. Instead, cortical A2AR and MT2R positively correlated to their respective ovarian analogs. The brain-ovarian crosstalk exits, even though the brain electrophysiology seemed more closely affected by ovarian hormones rather than cortical ones. We detected other weak correlations between EEG aspects and ovarian hormones, but we focused on the significant moderate-to-strong correlations.

Detecting a positive link between brain E2 and each ovarian E2 and A2AR highlighted the specific contribution of central E2 and ovarian E2 and A2AR in the brain-ovarian crosstalk. The link between brain and ovarian E2 harmonized the reported expression of brain estrogen receptors being modulated by gonadal steroids (Gillies & McArthur 2010). Apart from the extensively explored positive link between estrogen and adenosine signaling in gynecologic cancers and cancer chemotherapeutics (Lin et al. 2010; Mohamadi et al. 2018), such a link has been scarcely studied. For instance, in ovariectomized mice, the presence of low estrogen triggered myocardial injury, secondary to hyperactivity of adenosine-related signaling (Ndzie Noah et al. 2023). This study is the first to explore the estrogenic-adenosinergic link in psychopharmacology, using CAFF, AGO, and QUET.

The study limitations include the lack of progesterone, adenosine, and melatonin level measurements in the brain and ovaries. Nevertheless, using the current assessments, we could justify and interpret our findings.

In summary, our results indicated that the chronic consumption of CAFF was associated with reduced maximum EEG peak, slowed β wave, neurodegenerative changes, and reduced cortical E2Rα against increased A2AR immunoreactivity. Such changes highlighted the potential for cognitive deterioration and anti-epileptogenic activity. The addition of AGO or QUET to CAFF could not revert the EEG and microstructural changes; however, both AGO and QUET antagonized some of the CAFF-induced central endocrinal changes in terms of cortical E2Rα and A2AR. Both combinations added differential de novo EEG aspects, with the emergence of enhanced cortical MT2R immunoreactivity. Increased brain E2 was only obvious with QUET + CAFF. The EEG and the endocrinal alterations of the brain when AGO or QUET was co-administered with CAFF, especially when contrasted to AGO and QUET monotherapy, necessitates their future exploration as cognitive enhancers and anti-epileptogenic agents.

In the context of ovarian functions, and despite the disturbed ovarian microstructure observed with CAFF, the estrus cycle progression was not prominently affected. The CAFF-triggered hormonal disturbances of the ovarian milieu mimicked the cortical changes, apart from an additionally increased MT2R. The addition of AGO or QUET to CAFF did not revert ovarian microstructural disturbances with no obvious effects on the estrus cycle progression. Except for ovarian A2AR, the CAFF-associated ovarian hormonal derangements were not mitigated with the addition of either AGO or QUET. The brain–ovarian crosstalk was evident based on the significant correlations between some EEG aspects, cortical hormones, and the ovarian hormones. The contributions of female hormones, as well as adenosinergic and melatonergic signaling, were also evident.

Continued consumption of CAFF while taking AGO or QUET antagonized some of the CAFF-induced central and ovarian disturbances. Vigilance of female hormonal disturbances should be considered an integral part when advising patients on continued CAFF consumption with psychotropic medications, given the brain–ovarian mutual relationships. Further experimental studies and clinical trials remain warranted, with special emphasis on linking behavioral-to-ovarian function in terms of neurodegenerative disorders and psychopharmacology.

## Conflict of Interest

The authors declare that they have no conflict of interest.

## Supplementary Information

Below is the link to the electronic supplementary material.

Supplementary file1 (DOCX 4.00 MB)
