# Supplementary material for: Chronic Caffeine Consumption, Alone or Combined with Agomelatine or Quetiapine, Reduces the Maximum EEG Peak, As Linked to Cortical Neurodegeneration, Ovarian Estrogen Receptor Alpha, and Melatonin Receptor 2
Source: Psychopharmacology (Berl). 2024 Jun 6;241(10):2073–101. doi: 10.1007/s00213-024-06619-4 (PMC11442587; doi:10.1007/s00213-024-06619-4)
Supplement: Supplementary file 1 — Supplementary file1 (DOCX 4.00 MB) [file 213_2024_6619_MOESM1_ESM.docx]

**Chronic Caffeine Consumption, Alone or Combined with Agomelatine or Quetiapine, Reduces the Maximum EEG Peak, As Linked to Cortical Neurodegeneration, Ovarian Estrogen Receptor Alpha, and Melatonin Receptor 2**

**Sherine Abdelmissih^1^.** **Sara Adel Hosny^2^. Heba M. Elwi ^3^. Walaa Mohamed Sayed^4^. Mohamed Ali Eshra^5^. Olfat Gamil Shaker^3^. Nancy F. Samir^5^**

^1^Department of Medical Pharmacology, Faculty of Medicine Kasr Al-Ainy, Cairo University, Cairo, Egypt

^2^Department of Medical Histology, Faculty of Medicine Kasr Al-Ainy, Cairo University, Cairo, Egypt

^3^Department of Medical Biochemistry and Molecular Biology, Faculty of Medicine Kasr Al-Ainy, Cairo University, Cairo, Egypt

^4^Department of Anatomy and Embryology, Faculty of Medicine Kasr Al-Ainy, Cairo University, Cairo, Egypt

^5^Department of Medical Physiology, Faculty of Medicine Kasr Al-Ainy, Cairo University, Cairo, Egypt

**Correspondence:** Sherine Abdelmissih

e-mail: [drshery_wa@yahoo.com](mailto:drshery_wa@yahoo.com); [drshery_wa@kasralainy.edu.eg](mailto:drshery_wa@kasralainy.edu.eg)

**
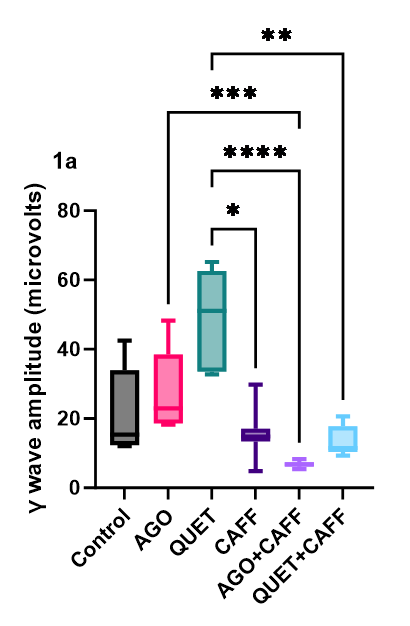

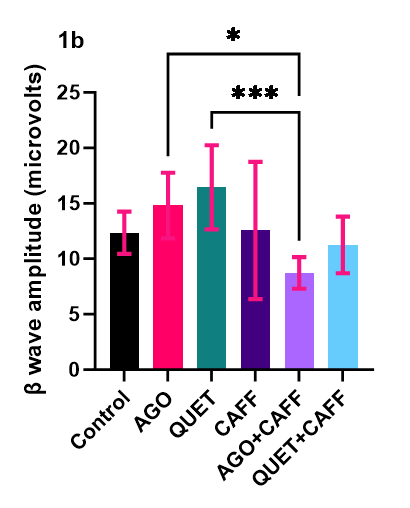
**

**
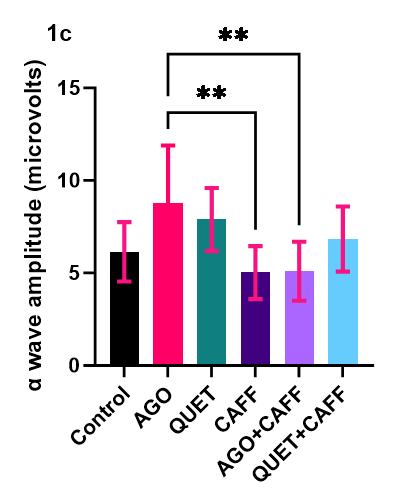

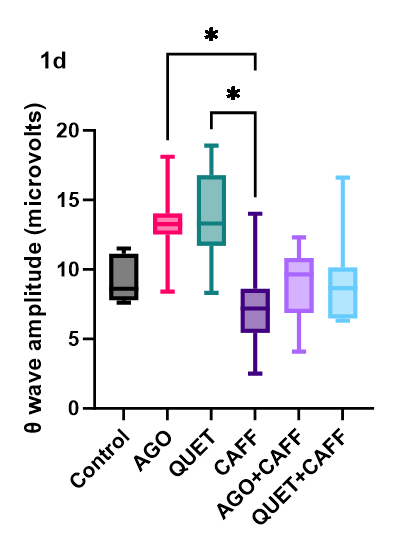
**

**Suppl. 1 EEG analysis of a)** γ amplitude; **b)** β amplitude; **c)** α amplitude; **d)** θ amplitude. Amplitudes (microvolts. **1a & 1d)** Data are analyzed using Kruskal–Wallis followed by *post hoc* Dunn’s test, and are represented as median and interquartile range. **1b & 1c)** Data are analyzed using ANOVA followed by *post hoc* Tukey’s test, and are represented as mean ± standard deviation (SD). *ρ* < 0.05*. *ρ* < 0.01**. *ρ* < 0.001***. *ρ* < 0.0001****. The graph is generated using Graph Pad Prism v.10.0.0. Adult female *Wistar* albino rats (N = 48) were equally subdivided into controls; AGO: 10 mg/kg agomelatine, oral, once daily; QUET: 10 mg/kg quetiapine, oral, once daily; CAFF: caffeine-containing beverages, as alternate-day coffee and cola, at room temperature, once daily; AGO + CAFF: caffeine-containing beverages followed by 10 mg/kg agomelatine, oral, once daily; QUET + CAFF: caffeine-containing beverages followed by 10 mg/kg quetiapine, oral, once daily. All administrations were adopted for 8 weeks.


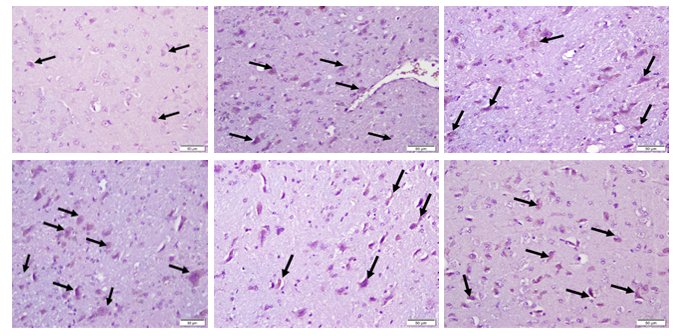


**Suppl. 2** **Photomicrographs of E2Rα immunoreactive area in rat cerebral cortex**. From left to right over two rows, **a.** Control group (×200). **b.** AGO (×200). **c.** QUET (×200). **d.** CAFF (×200). **e.** AGO + CAFF (×200). **f.** QUET + CAFF (×200). The E2Rα-immunoreactive area is marked by black arrows. E2Rα: estrogen receptor alpha. Adult female *Wistar* albino rats (N = 48) were equally subdivided into controls; AGO: 10 mg/kg agomelatine, oral, once daily; QUET: 10 mg/kg quetiapine, oral, once daily; CAFF: caffeine-containing beverages, as alternate-day coffee and cola, at room temperature, once daily; AGO + CAFF: caffeine-containing beverages followed by 10 mg/kg agomelatine, oral, once daily; QUET + CAFF: caffeine-containing beverages followed by 10 mg/kg quetiapine, oral, once daily. All administrations were adopted for 8 weeks.

**
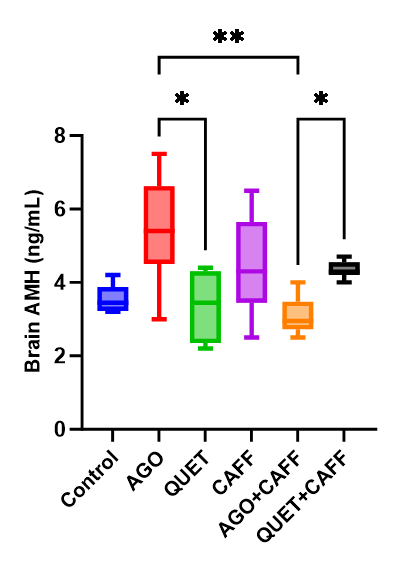
**

**Suppl. 3 Brain AMH (ng/mL).** Data are analyzed using Kruskal–Wallis followed by *post hoc* Dunn’s test, and are represented as median and interquartile range. *ρ* < 0.05*; *ρ* < 0.001***. The graph is generated using Graph Pad Prism v.10.0.0. AMH: antimullerian hormone. Adult female *Wistar* albino rats (N = 48) were equally subdivided into controls; AGO-treated: 10 mg/kg agomelatine, oral, once daily; QUET-treated: 10 mg/kg quetiapine, oral, once daily; CAFF: caffeine-containing beverages, as alternate-day coffee and cola, at room temperature, once daily; AGO + CAFF: caffeine-containing beverages followed by 10 mg/kg agomelatine, oral, once daily; QUET + CAFF: caffeine-containing beverages followed by 10 mg/kg quetiapine, oral, once daily. All administrations were given for 8 weeks.


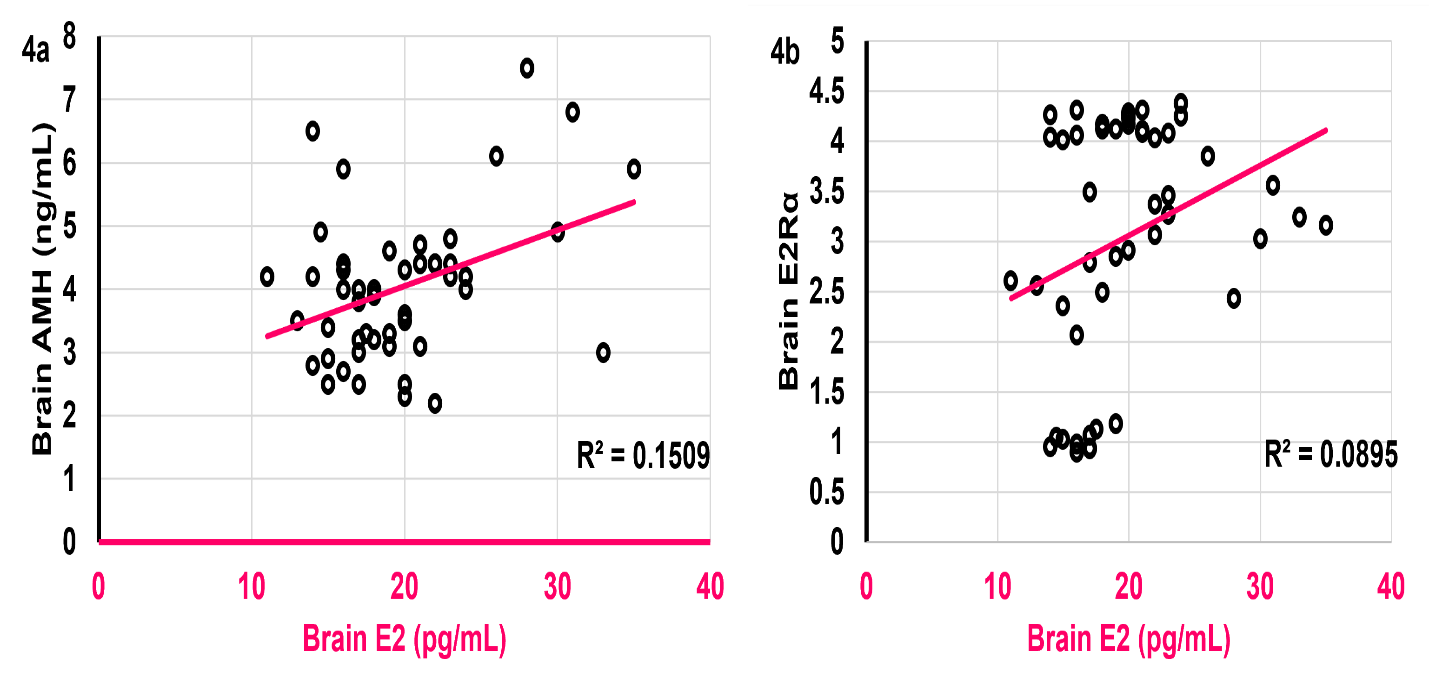


**Suppl. 4 Scatter plots illustrating correlations between a.** Brain E2 and brain AMH. **b.** Brain E2 and cortical E2Rα**.** Scatter plots are generated using Microsoft Excel (Microsoft Office 365). Spearman *rho* correlation. Significant when *ρ* < 0.05. E2: estradiol; AMH: antimullerian hormone; E2Rα: estrogen receptor alpha. Adult female *Wistar* albino rats (N = 48) were equally subdivided into controls; AGO: 10 mg/kg agomelatine, oral, once daily; QUET: 10 mg/kg quetiapine, oral, once daily; CAFF: caffeine-containing beverages, as alternate-day coffee and cola, at room temperature, once daily; AGO + CAFF: caffeine-containing beverages followed by 10 mg/kg agomelatine, oral, once daily; QUET + CAFF: caffeine-containing beverages followed by 10 mg/kg quetiapine, oral, once daily. All administrations were adopted for 8 weeks.


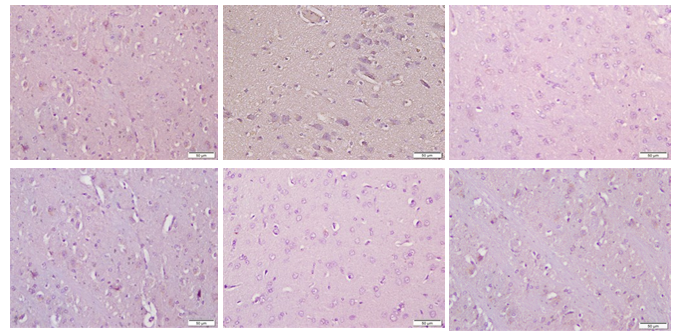


**Suppl. 5** **Photomicrographs of A2AR immunoreactivity in rat cerebral cortex**. From left to right over two rows, **a.** Control (×200). **b.** AGO (×200). **c.** QUET (×200). **d.** CAFF (×200). **e.** AGO + CAFF (×200). **f.** QUET + CAFF (×200). A2AR immunoreactivity is shown as darker purple dots. A2AR: adenosine receptor 2A. Rats (N = 48) were equally subdivided into controls; AGO: 10 mg/kg agomelatine, oral, once daily; QUET: 10 mg/kg quetiapine, oral, once daily; CAFF: caffeine-containing beverages, as alternate-day coffee and cola, at room temperature, once daily; AGO + CAFF: caffeine-containing beverages followed by 10 mg/kg agomelatine, oral, once daily; QUET + CAFF: caffeine-containing beverages followed by 10 mg/kg quetiapine, oral, once daily. All administrations were adopted for 8 weeks.


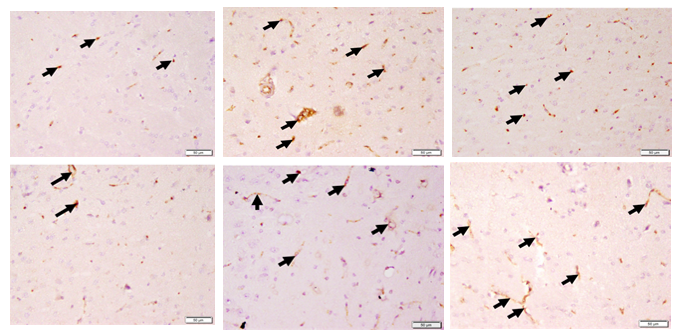


**Suppl. 6** **Photomicrographs of MT2R immunoreactive cells in rat cerebral cortex**. As ordered from left to right over two rows, **a.** Control (×200). **b.** AGO (×200). **c.** QUET (×200). **d.** CAFF (×200). **e.** AGO + CAFF (×200). **f.** QUET + CAFF (×200). MT2R immunoreactive pyramidal cells are indicated by black arrows, and the MT2R immunoreactive cytoplasm of pyramidal cells is indicated by a brownish color. MT2R: melatonin receptor 2. Adult female *Wistar* albino rats (N = 48) were equally subdivided into controls; AGO: 10 mg/kg agomelatine, oral, once daily; QUET: 10 mg/kg quetiapine, oral, once daily; CAFF: caffeine-containing beverages, as alternate-day coffee and cola, at room temperature, once daily; AGO + CAFF: caffeine-containing beverages followed by 10 mg/kg agomelatine, oral, once daily; QUET + CAFF: caffeine-containing beverages followed by 10 mg/kg quetiapine, oral, once daily. All administrations were adopted for 8 weeks.


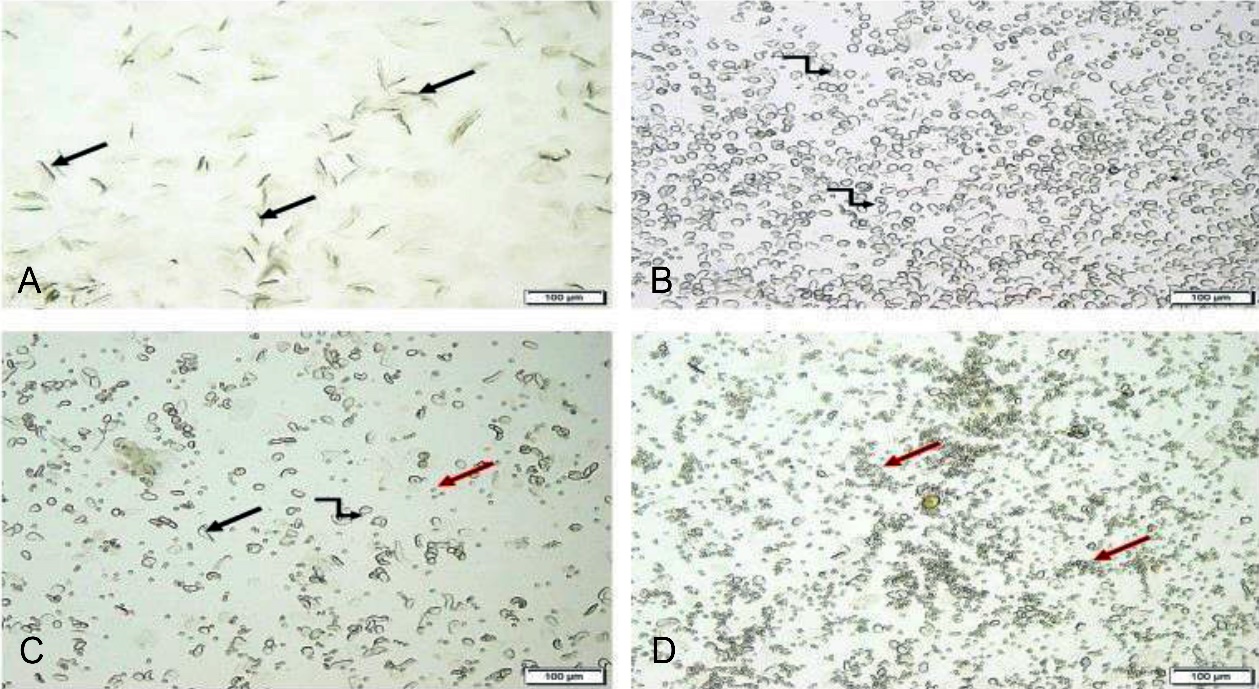


**Suppl. 7 Four phases of the rat estrous cycle. a.** The proestrus phase, with evident nucleated epithelial cells (black arrows), corresponds to the human follicular stage (increased circulating estradiol). **b.** The estrus phase, which is characterized by the presence of non-nucleated cells (kinked arrows), corresponds to ovulation (FSH peak, LH surge, and a rapid decline in estradiol). **c.** The metestrus phase, with lymphocytes (red arrow), epithelial cells (black arrow), and non-nucleated cells (kinked arrow), corresponds to the early secretory phase (increased progesterone). **d.** The diestrus phase, with numerous lymphocytes (red arrows), corresponds to the late secretory phase (increased progesterone). Unstained vaginal smears of adult female *Wistar* albino rats, visualized under light microscope.


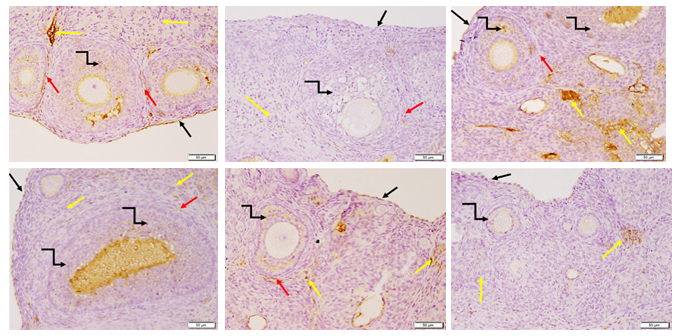


**Suppl. 8** **Photomicrographs of** **E2Rα immunoreactive area in rat ovaries**. From left to right over two rows, **a.** Control group (×200), **b.** AGO (×200), **c.** QUET (×200), **d.** CAFF (×200), **e.** AGO + CAFF (×200), and **f.** QUET + CAFF (×200). E2Rα immunoreactive, ovarian epithelial cells (black arrows), granulosa cells (kinked arrows), theca cells (red arrows), and cortical stromal cells (yellow arrows). E2Rα: estrogen receptor alpha. Adult female *Wistar* albino rats (N = 48) were equally subdivided into controls; AGO: 10 mg/kg agomelatine, oral, once daily; QUET: 10 mg/kg quetiapine, oral, once daily; CAFF: caffeine-containing beverages, as alternate-day coffee and cola, at room temperature, once daily; AGO + CAFF: caffeine-containing beverages followed by 10 mg/kg agomelatine, oral, once daily; QUET + CAFF: caffeine-containing beverages followed by 10 mg/kg quetiapine, oral, once daily. All administrations were adopted for 8 weeks.


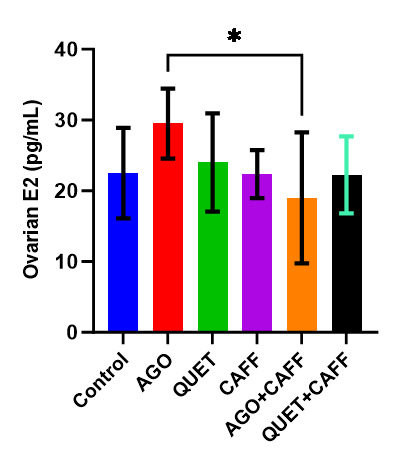


**Suppl. 9** **Ovarian E2 (pg/mL).** Data are analyzed using ANOVA followed by *post hoc* Tukey’s test and are represented as mean ± standard deviation (SD). *ρ* < 0.05*. The graph is generated using Graph Pad Prism v.10.0.0. E2: estradiol. Adult female *Wistar* albino rats (N = 48) were equally subdivided into controls; AGO: 10 mg/kg agomelatine, oral, once daily; QUET: 10 mg/kg quetiapine, oral, once daily; CAFF: caffeine-containing beverages, as alternate-day coffee and cola, at room temperature, once daily; AGO + CAFF: caffeine-containing beverages followed by 10 mg/kg agomelatine, oral, once daily; QUET + CAFF: caffeine-containing beverages followed by 10 mg/kg quetiapine, oral, once daily. All administrations were adopted for 8 weeks.


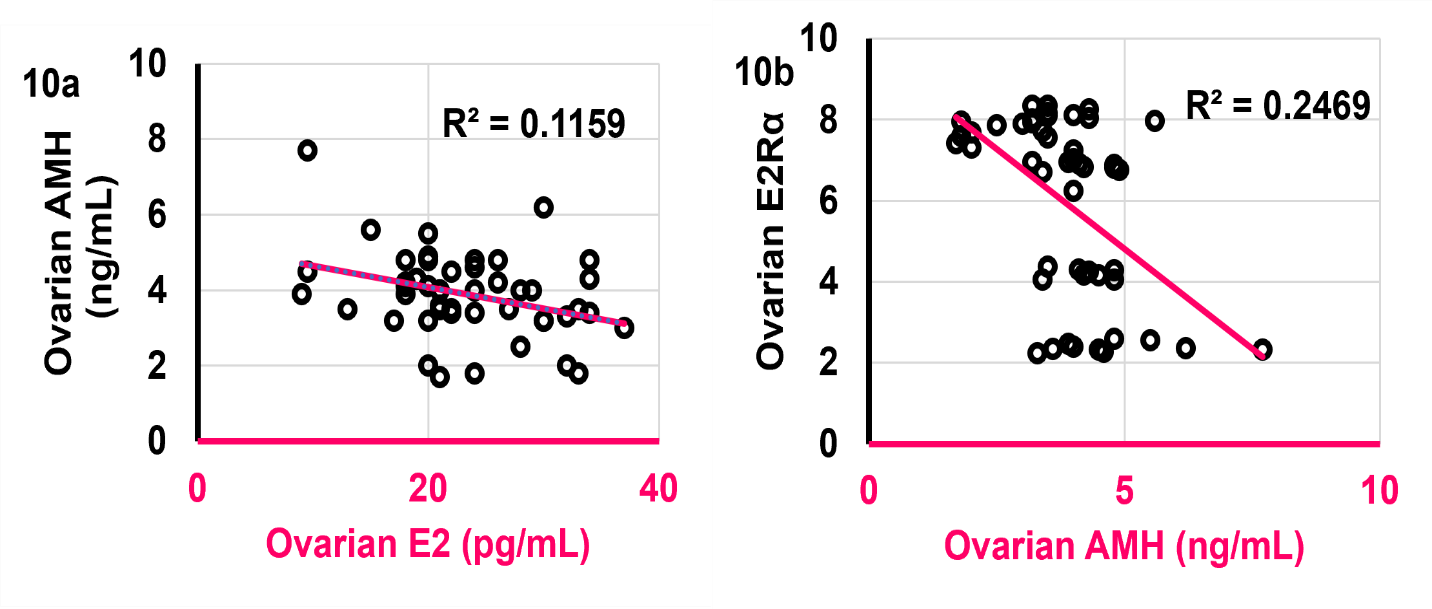


**Suppl. 10 Scatter plots illustrating correlations between a.** ovarian E2 (pg/mL) and ovarian AMH (ng/mL). **b.** Ovarian AMH (ng/mL) and ovarian E2Rα. Scatter plots are generated using Microsoft Excel (Microsoft Office 365). Spearman *rho* correlation. *ρ* < 0.05 indicated significant different. E2: estradiol; AMH: antimullerian hormone; E2Rα: estrogen receptor alpha. Adult female *Wistar* albino rats (N = 48) were equally subdivided into controls; AGO: 10 mg/kg agomelatine, oral, once daily, QUET: 10 mg/kg quetiapine, oral, once daily; CAFF: caffeine-containing beverages, as alternate-day coffee and cola, at room temperature, once daily; AGO + CAFF: caffeine-containing beverages followed by 10 mg/kg agomelatine, oral, once daily; QUET + CAFF: caffeine-containing beverages followed by 10 mg/kg quetiapine, oral, once daily. All administrations were adopted for 8 weeks.


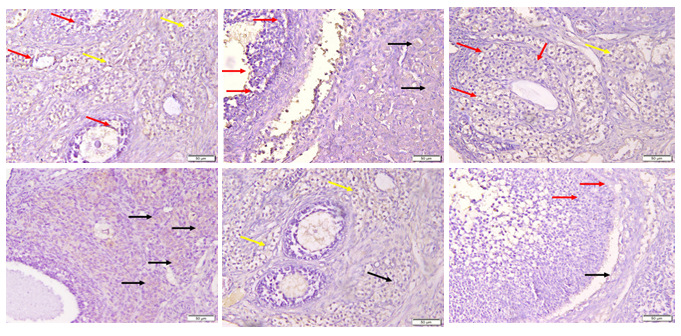


**Suppl. 11** **Photomicrographs of** **A2AR immunoreactive area in rat ovaries**. From left to right over two rows, **a.** control, exhibiting multiple follicles with A2AR immunoreactive cytoplasm of granulosa cells (red arrows) and A2AR immunoreactive membrane of cortical stromal cells (yellow arrows) (×200). **b.** AGO, exhibiting large follicles with A2AR immunoreactive membranes of granulosa cells (red arrows) and luteal cells (black arrows) (×200). **c.** QUET, showing A2AR immunoreactive membrane of granulosa cells (red arrows) and cortical stromal cells (yellow arrow) (×200). **d.** CAFF, exhibiting multiple follicles with A2AR immunoreactive cytoplasm of luteal cells (black arrows) (×200), **e.** AGO+CAFF, exhibiting ovarian corpora with A2AR immunoreactive cytoplasm of granulosa-lutein cells (black arrow) and A2AR immunoreactive membrane of cortical stromal cells (yellow arrows) (×200). **f.** QUET + CAFF, exhibiting A2AR immunoreactive cytoplasm of granulosa cells (red arrows) and theca cells (black arrow) (×200). A2AR: adenosine receptor 2A. Adult female *Wistar* albino rats (N = 48) were equally subdivided into controls; AGO: 10 mg/kg agomelatine, oral, once daily; QUET: 10 mg/kg quetiapine, oral, once daily; CAFF: caffeine-containing beverages, as alternate-day coffee and cola, at room temperature, once daily; AGO + CAFF: caffeine-containing beverages followed by 10 mg/kg agomelatine, oral, once daily; QUET + CAFF: caffeine-containing beverages followed by 10 mg/kg quetiapine, oral, once daily. All administrations were adopted for 8 weeks.


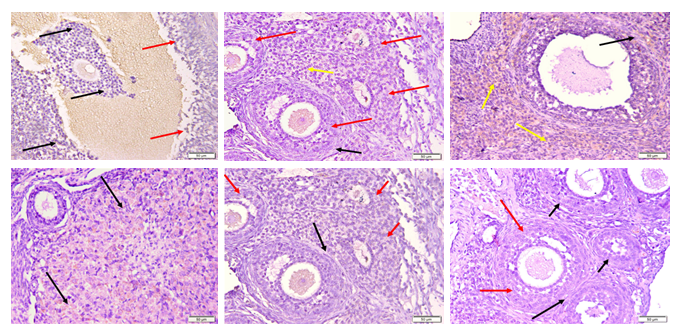


**Suppl. 12** **Photomicrographs of MT2R- immunoreactive area in rat ovaries**. From left to right over two rows, **a.** control, exhibiting mature Graafian follicle with MT2R immunoreactive cytoplasm of granulosa cells (red arrows) and theca cells (black arrows) (×200). **b.** AGO, exhibiting large follicles with MT2R immunoreactive cytoplasm of granulosa cells (red arrows), theca cells (black arrow), and cortical stromal cells (yellow arrow) (×200). **c.** QUET, showing MT2R immunoreactive theca cells (black arrow) and cortical stroma cells (yellow arrows) (×200). **d.** CAFF, exhibiting MT2R immunoreactive theca cells (black arrows) (×200). **e.** AGO + CAFF, exhibiting ovarian corpus with MT2R immunoreactive granulosa cells (red arrows) and theca cells (black arrow) (×200). **f.** QUET + CAFF, exhibiting MT2R immunoreactive cytoplasm of granulosa cells (red arrows) and theca cells (black arrows) (×200). MT2R: melatonin receptor 2. Adult female *Wistar* albino rats (N = 48) were equally subdivided into controls; AGO: 10 mg/kg agomelatine, oral, once daily; QUET: 10 mg/kg quetiapine, oral, once daily; CAFF: caffeine-containing beverages, as alternate-day coffee and cola, at room temperature, once daily; AGO + CAFF: caffeine-containing beverages followed by 10 mg/kg agomelatine, oral, once daily; QUET + CAFF: caffeine-containing beverages followed by 10 mg/kg quetiapine, oral, once daily. All administrations were adopted for 8 weeks.

**Suppl. 13. Summary of the findings, with emphasis on significant differences compared with control and CAFF.**

| **Groups**  **Assessment** | **CAFF** | **AGO+CAFF** | **QUET+CAFF** | | | **AGO** | **QUET** |
| --- | --- | --- | --- | --- | --- | --- | --- |
| **Maximum EEG peak** | Reduced ^a^ | | | | |  | |
| **β wave frequency** | Slower **^a^** (within β range) |  | | | | | |
| **TTP** |  | Delayed **^a^** |  | | | | |
| **δ wave frequency** |  | Faster **^a b^** (within δ range) |  | | | | |
| **Source EEG frequency** |  | | Slower **^a^** (to δ range) | | |  | |
| **δ wave amplitude** |  | | Reduced **^b^** | | |  | |
| **Source EEG amplitude** |  | | | | | | Increased **^a b^** |
| **γ wave frequency** |  | | | | | | Slower **^a b^** (to β range) |
| **Brain microstructure** | Degenerated cells | | | | |  | Degenerated cells |
|  | Dilated congested blood vessels | | | | |  | |
|  | Multinucleated cells |  | Multinucleated cells | | |  | |
|  | Glial cells |  | Glial cells | | | | |
|  | Pyknotic cells |  | Pyknotic cells | | |  | |
| **Number of degenerated pyramidal cells** | Increased **^a^** | | | | |  | |
| **Brain E2** |  | | Increased **^a b^** | | | |  |
| **Brain AMH** |  | | | | | | |
| **Cortical E2Rα** | Decreased ^a^ | Increased ^a b^ | | | Increased ^a^ | | |
| **Cortical A2AR** | Increased **^a^** | Redeemed | | | | Deceased **^a^** | |
| **Cortical MT2R** |  | Increased **^a^** | | | |  | |
| **Estrus cycle progression** | No significant delay | | | | | | |
| **Ovarian microstructure** | Cystic follicles with atretic oocytes | | | | |  | |
|  | Congested blood vessels |  | | | | | Congested blood vessels |
|  |  | Large corpus luteum | | | |  | |
| **Ovarian E2** |  | | | | | | |
| **Ovarian AMH** |  | Increased **^a^** |  | | | | |
| **Ovarian E2Rα** | Decreased **^a^** | | | | |  | Decreased **^a^** |
| **Ovarian A2AR** | Increased ^a^ | Redeemed | Decreased ^a^ | | | | |
| **Ovarian MT2R** | Increased **^a^** | Increased **^a b^** | | Increased **^a^** | | | |

Rats (N = 48) were equally subdivided into 6 groups: control, AGO (10 mg/kg), QUET (10 mg/kg), CAFF group (coffee and cola on alternate days), AGO+CAFF (CAFF followed by 10 mg/kg AGO), and QUET+CAFF (CAFF followed by 10 mg/kg QUET). All treatments were administered orally, once daily, for 8 weeks. Estrus cycle progression was tracked. EEG was conducted at the end of study. Rats were euthanized, followed by histologic examination of the brain and ovaries, biochemical analysis of brain and ovarian E2 and AMH levels, and immunohistochemistry of cortical and ovarian E2Rα, A2AR, and MT2R. significant when *ρ<* 0.05 compared with ^a^ the control and ^b^ CAFF. AGO: agomelatine; QUET: quetiapine; CAFF: caffeine-containing beverages; EEG: electroencephalography; TTP: time-to-peak; E2: estradiol; AMH: antimullerian hormone; E2Rα: estrogen receptor alpha; A2AR: adenosine receptor 2A; MT2R: melatonin receptor 2.
